# A multi-disciplinary perspective on emergent and future innovations in peer review

**DOI:** 10.12688/f1000research.12037.3

**Published:** 2017-11-29

**Authors:** Jonathan P. Tennant, Jonathan M. Dugan, Daniel Graziotin, Damien C. Jacques, François Waldner, Daniel Mietchen, Yehia Elkhatib, Lauren B. Collister, Christina K. Pikas, Tom Crick, Paola Masuzzo, Anthony Caravaggi, Devin R. Berg, Kyle E. Niemeyer, Tony Ross-Hellauer, Sara Mannheimer, Lillian Rigling, Daniel S. Katz, Bastian Greshake Tzovaras, Josmel Pacheco-Mendoza, Nazeefa Fatima, Marta Poblet, Marios Isaakidis, Dasapta Erwin Irawan, Sébastien Renaut, Christopher R. Madan, Lisa Matthias, Jesper Nørgaard Kjær, Daniel Paul O'Donnell, Cameron Neylon, Sarah Kearns, Manojkumar Selvaraju, Julien Colomb

**Affiliations:** 1 ScienceOpen, Berlin, Germany; 2 Imperial College London, London, UK; 3 Berkeley Institute for Data Science, University of California, Berkeley, CA, USA; 4 Institute of Software Technology, University of Stuttgart, Stuttgart, Germany; 5 Earth and Life Institute, Université catholique de Louvain, Louvain-la-Neuve, Belgium; 6 Data Science Institute, University of Virginia, Charlottesville, VA, USA; 7 School of Computing and Communications, Lancaster University, Lancaster, UK; 8 University Library System, University of Pittsburgh, Pittsburgh, PA, USA; 9 Johns Hopkins University Applied Physics Laboratory, Laurel, MD, USA; 10 Cardiff Metropolitan University, Cardiff, UK; 11 Department of Biochemistry, Ghent University, Ghent, Belgium; 12 VIB-UGent Center for Medical Biotechnology, Ghent, Belgium; 13 School of Biological, Earth and Environmental Sciences, University College Cork, Cork, Ireland; 14 Engineering & Technology Department, University of Wisconsin-Stout, Menomonie, WI, USA; 15 School of Mechanical, Industrial, and Manufacturing Engineering, Oregon State University, Corvallis, OR, USA; 16 State and University Library, University of Göttingen, Göttingen, Germany; 17 Montana State University, Bozeman, MT, USA; 18 Western University Libraries, London, ON, USA; 19 School of Information Sciences, University of Illinois at Urbana-Champaign, Urbana, IL, USA; 20 Department of Electrical and Computer Engineering, University of Illinois Urbana-Champaign, Urbana, IL, USA; 21 Department of Computer Science, University of Illinois Urbana-Champaign, Urbana, IL, USA; 22 National Center for Supercomputing Applications, University of Illinois at Urbana-Champaign, Urbana, IL, USA; 23 Institute of Cell Biology and Neuroscience, Goethe University Frankfurt, Frankfurt, Germany; 24 Universidad San Ignacio de Loyola, Lima, Peru; 25 Department of Biology, Faculty of Science, Lund University, Lund, Sweden; 26 Graduate School of Business and Law, RMIT University, Melbourne, Australia; 27 Department of Computer Science, University College London, London, UK; 28 Department of Groundwater Engineering, Faculty of Earth Sciences and Technology, Institut Teknologi Bandung, Bandung, Indonesia; 29 Département de Sciences Biologiques, Institut de Recherche en Biologie Végétale, Université de Montréal, Montreal, QC, Canada; 30 School of Psychology, University of Nottingham, Nottingham, UK; 31 OpenAIRE, University of Göttingen, Göttingen, Germany; 32 Department of Affective Disorders, Psychiatric Research Academy, Aarhus University Hospital, Risskov, Denmark; 33 Department of English and Centre for the Study of Scholarly Communications, University of Lethbridge, Lethbridge, AB, Canada; 34 Centre for Culture and Technology, Curtin University, Perth, Australia; 35 Department of Chemical Biology, University of Michigan, Ann Arbor, MI, USA; 36 Integrated Gulf Biosystems, Riyadh, Saudi Arabia; 37 Saudi Human Genome Program, King Abdulaziz City for Science and Technology (KACST), Riyadh, Saudi Arabia; 38 Independent Researcher, Berlin, Germany

**Keywords:** Open Peer Review, Social Media, Web 2.0, Open Science, Scholarly Publishing, Incentives, Quality Control

## Abstract

Peer review of research articles is a core part of our scholarly communication system. In spite of its importance, the status and purpose of peer review is often contested. What is its role in our modern digital research and communications infrastructure? Does it perform to the high standards with which it is generally regarded? Studies of peer review have shown that it is prone to bias and abuse in numerous dimensions, frequently unreliable, and can fail to detect even fraudulent research. With the advent of web technologies, we are now witnessing a phase of innovation and experimentation in our approaches to peer review. These developments prompted us to examine emerging models of peer review from a range of disciplines and venues, and to ask how they might address some of the issues with our current systems of peer review. We examine the functionality of a range of social Web platforms, and compare these with the traits underlying a viable peer review system: quality control, quantified performance metrics as engagement incentives, and certification and reputation. Ideally, any new systems will demonstrate that they out-perform and reduce the biases of existing models as much as possible. We conclude that there is considerable scope for new peer review initiatives to be developed, each with their own potential issues and advantages. We also propose a novel hybrid platform model that could, at least partially, resolve many of the socio-technical issues associated with peer review, and potentially disrupt the entire scholarly communication system. Success for any such development relies on reaching a critical threshold of research community engagement with both the process and the platform, and therefore cannot be achieved without a significant change of incentives in research environments.

## 1 Introduction

Peer review is a core part of our self-regulating global scholarship system. It defines the process in which professional experts (peers) are invited to critically assess the quality, novelty, theoretical and empirical validity, and potential impact of research by others, typically while it is in the form of a manuscript for an article, conference, or book ( Daniel, 1993; Kronick, 1990; Spier, 2002; Zuckerman & Merton, 1971). For the purposes of this article, we are exclusively addressing peer review in the context of manuscript selection for scientific research articles, with some initial considerations of other outputs such as software and data. In this form, peer review is becoming increasingly central as a principle of mutual control in the development of scholarly communities that are adapting to digital, information-rich, publishing-driven research ecosystems. Consequently, peer review is a vital component at the core of research communication processes, with repercussions for the very structure of academia, which largely operates through a peer reviewed publication-based reward and incentive system ( Moore *et al.*, 2017). Different forms of peer review beyond that for manuscripts are also clearly important and used in other contexts such as academic appointments, measurement time, research ethics or research grants (see, e.g., Fitzpatrick, 2011b, p. 16), but a holistic discussion of all forms of peer review is beyond the scope of the present article.

Peer review is not a singular or static entity. It comes in various flavors that result from different approaches to the relative timing of the review in the publication cycle, the reciprocal transparency of the process, and the contrasting and disciplinary practices ( Ross-Hellauer, 2017). Such interdisciplinary differences have made the study and understanding of peer review highly complex, and implementing any systemic changes to peer review is fraught with the challenges of synchronous adoption between heterogeneous communities often with vastly different social norms and practices. The criteria used for evaluation, including methodological soundness or expected scholarly impact, are typically important variables to consider, and again vary substantially between disciplines. However, peer review is still often perceived as a “gold standard” of scholarly communication (e.g., D’Andrea & O’Dwyer (2017); Mayden (2012)), despite the inherent diversity of the process and never an original intention to be used as such. Peer review is a diverse method of quality control, and applied inconsistently both in theory and practice ( Casnici *et al.*, 2017; Pontille & Torny, 2015), and generally lacks any form of transparency or formal standardization. As such, it remains difficult to know precisely what a “peer reviewed publication” means.

Traditionally, the function of peer review has been as a vetting procedure or gatekeeper to assist the distribution of limited resources—for instance, space in peer reviewed print publication venues. With the advent of the internet, the physical constraints on distribution are no longer present, and, at least in theory, we are now able to disseminate research content rapidly and at relatively negligible cost ( Moore *et al.*, 2017). This has led to the innovation and increasing popularity of digital-only publication venues that vet submissions based exclusively on the soundness of the research, often termed “mega-journals” (e.g., *PLOS ONE*, *PeerJ*, the *Frontiers* series). Such a flexibility in the filter function of peer review reduces, but does not eliminate, the role of peer review as a selective gatekeeper, and can be considered to be “impact neutral.” Due to such digital experimentations, ongoing discussions about peer review are intimately linked with contemporaneous developments in Open Access (OA) publishing and to broader changes in open scholarship ( Tennant *et al.*, 2016).

The goal of this article is to investigate the historical evolution in the theory and application of peer review in a socio-technological context. We use this as the basis to consider how specific traits of consumer social Web platforms can be combined to create an optimized hybrid peer review model that we suggest will be more efficient, democratic, and accountable than existing processes.

### 1.0.1 Methods

This article provides a general review of conventional journal article peer review and evaluation of recent and current innovations in the field. It is not a systematic review or meta-analysis of the empirical literature (i.e., we did not perform a formal search strategy undertaken with specific keywords). Rather, a team of researchers with diverse expertise in the sciences, scholarly publishing and communication, and libraries pooled their knowledge to collaboratively and iteratively analyze and report on the present literature and current innovations. The reviewed and cited articles within were identified and selected through searches of general research databases (e.g., *Web of Science*, *Google Scholar*, and *Scopus*) as well as specialized research databases (e.g., *Library & Information Science Abstracts* (LISA) and *PubMed*). Particularly relevant articles were used to seed identification of cited, citing, and articles related by citation. The team co-ordinated efforts using an online collaboration tool ( *Slack*) to share, discuss, debate, and come to consensus. Authoring and editing was also done collaboratively and in public view using *Overleaf*. Each co-author independently contributed original content and participated in the reviewing, editing and discussion process.

### 1.1 The history and evolution of peer review

Any discussion on innovations in peer review must appreciate its historical context. By understanding the history of scholarly publishing and the interwoven evolution of peer review, we recognize that neither are static entities, but covary with each other. By learning from historical experiences, we can also become more aware of how to shape future directions of peer review evolution and gain insight to what the process should look like in an optimal world. The actual term “peer review” only appears in the scientific press in the 1960s. Even in the 1970s, it was often associated with grant review and not with evaluation and selection for publishing ( Baldwin, 2017a). However, the history of evaluation and selection processes for publication clearly predates the 1970s.

**Figure 1.**
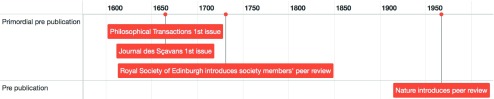
A brief timeline of the evolution of peer review: The primordial times. The interactive data visualization is available at https://dgraziotin.shinyapps.io/peerreviewtimeline, and the source code and data are available at https://doi.org/10.6084/m9.figshare.5117260

***1.1.1 The early history of peer review.*** The origins of a form of “peer review” for scholarly research articles are commonly associated with the formation of national academies in 17th century Europe, although some have found foreshadowing of the practice ( Al-Rahawi, c900; Csiszar, 2016; Fyfe *et al.*, 2017; Spier, 2002). We call this period the *primordial* time of peer review ( Figure 1), but note that the term “peer review” was not formally used then. Biagioli (2002) described in detail the gradual differentiation of peer review from book censorship, and the role that state licensing and censorship systems played in 16th century Europe; a period when monographs were the primary mode of communication. Several years after the Royal Society of London (1660) was established, it created its own in-house journal, *Philosophical Transactions*. Around the same time, Denis de Sallo published the first issue of *Journal des Sçavans*, and both of these journals were first published in 1665 ( Manten, 1980; Oldenburg, 1665; Zuckerman & Merton, 1971). With this origin, early forms of peer evaluation emerged as part of the social practices of gentlemanly learned societies ( Kronick, 1990; Moxham & Fyfe, 2017; Spier, 2002). The development of these prototypical scientific journals gradually replaced the exchange of experimental reports and findings through correspondence, formalizing a process that had been essentially personal and informal until then. “Peer review”, during this time, was more of a civil, collegial discussion in the form of letters between authors and the publication editors ( Baldwin, 2017b). Social pressures of generating new audiences for research, as well as new technological developments such as the steam-powered press, were also crucial ( Shuttleworth & Charnley, 2016). From these early developments, the process of independent review of scientific reports by acknowledged experts, besides the editors themselves, gradually emerged ( Csiszar, 2016). However, the review process was more similar to non-scholarly publishing, as the editors were the only ones to appraise manuscripts before printing ( Burnham, 1990). The primary purpose of this process was to select information for publication to account for the limited distribution capacity, and remained the authoritative purpose of such evaluation for more than two centuries.

***1.1.2 Adaptation through commercialisation.*** Peer review in forms that we would now recognize emerged in the early 19th century due to the increasing professionalism of science, and primarily through English scholarly societies. During the 19th century, there was a proliferation of scientific journals, and the diversity, quantity, and specialization of the material presented to journal editors increased. Peer evaluations evolved to become more about judgements of scientific integrity, but the intention of any such process was never for the purposes of gate-keeping ( Csiszar, 2016). Research diversification made it necessary to seek assistance outside the immediate group of knowledgeable reviewers from the journals’ sponsoring societies ( Burnham, 1990). Evaluation evolved to become a largely outsourced process, which still persists in modern scholarly publishing today. The current system of formal peer review, and use of the term itself, only emerged in the mid-20th century in a very piecemeal fashion (and in some disciplines, the late 20th century or early 21st; see Graf, 2014, for an example of a major philological journal which began systematic peer review in 2011). *Nature*, now considered a top journal, did not initiate any sort of peer review process until at least 1967, only becoming part of the formalised process in 1973 ( nature.com/nature/history/timeline_1960s.html).

This editor-led process of peer review became increasingly mainstream and important in the post-World War II decades, and is what we term “traditional” or “conventional” peer review throughout this article. Such expansion was primarily due to the development of a modern academic prestige economy based on the perception of quality or excellence surrounding journal-based publications ( Baldwin, 2017a; Fyfe *et al.*, 2017). Peer review increasingly gained symbolic capital as a process of objective judgement and consensus. The term itself became formalised in research processes, borrowed from government bodies who employed it for aiding selective distribution of research funds ( Csiszar, 2016). The increasing professionalism of academies enabled commercial publishers to use peer review as a way of legitimizing their journals ( Baldwin, 2015; Fyfe *et al.*, 2017), and capitalized on the traditional perception of peer review as voluntary duty by academics to provide these services. A consequence of this was that peer review became a more homogenized process that enabled private publishing companies to thrive, and eventually establish a dominant, oligopolistic marketplace position ( Larivière *et al.*, 2015). This represented a shift from peer review as a more synergistic activity among scholars, to commercial entities selling it as an added value service back to the same academic community who was performing it freely for them. The estimated cost of peer review is a minimum of £1.9bn per year (in 2008; Research Information Network (2008)), representing a substantial vested financial interest in maintaining the current process of peer review ( Smith, 2010). Neither account for overhead costs in publisher management, or the redundancy of the reject-resubmit cycle authors enter due to the competition for the symbolic value of journal prestige ( Jubb, 2016).

The result of this is that modern peer review has become enormously complicated. By allowing the process to become managed by a hyper-competitive publishing industry and integrated with academic career progression, developments in scholarly communication have become strongly coupled to the transforming nature of academic research institutes. These institutes have now evolved into internationally competitive businesses that strive for impact through journal publication. Often this is now mediated by commercial publishers through attempts to align their products with the academic ideal of research excellence ( Moore *et al.*, 2017). Such a consequence is plausibly related to, or even a consequence of, broader shifts towards a more competitive neoliberal academic culture ( Raaper, 2016). Here, emphasis is largely placed on production and standing, value, or utility ( Gupta, 2016), as opposed to the original primary focus of research on discovery and novelty.

**Figure 2.**
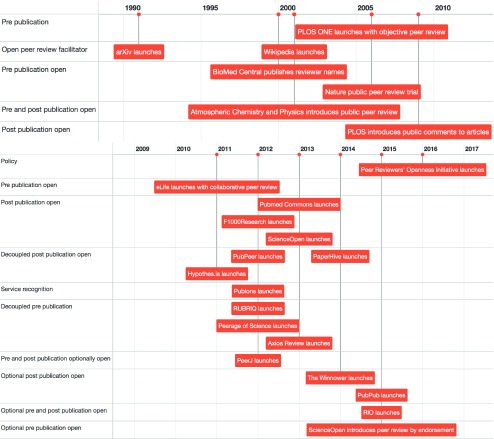
A brief timeline of the evolution of peer review: The revolution. See text for more details on individual initiatives. The interactive data visualization is available at https://dgraziotin.shinyapps.io/peerreviewtimeline, and the source code and data are available at https://doi.org/10.6084/m9.figshare.5117260

***1.1.3 The peer review revolution.*** In the last several decades, and boosted by the emergence of Web-based technologies, there have been substantial innovative efforts to decouple peer review from the publishing process ( Figure 2; Schmidt & Görögh (2017)), and the ever increasing volume of published research. Much of this experimentation has been based on earlier precedents, and in some cases a total reversal back to historical processes. Such decoupling attempts have typically been achieved by adopting peer review as an overlay process on top of formally published research articles, or by pursuing a “publish first, filter later” protocol, with peer review taking place after the initial publication of research results ( BioMed Central, 2017; McKiernan *et al.*, 2016; Moed, 2007). Here, the meaning of “publication” becomes “making public,” as in the legal and common use as opposed to the scholarly publishing sense where it also implies peer reviewed, a trait unique to research scholarship. In fields such as Physics, Mathematics, and Economics, it is common for authors to send their colleagues either paper or electronic copies of their manuscripts for pre-submission evaluation. Launched in 1991, *arXiv* ( arxiv.org) formalized this process by creating a central network for whole communities to access such e-prints. Today, *arXiv* has more than one million e-prints from various research fields and receives more than 8,000 monthly submissions ( arXiv, 2017). Here, e-prints or preprints are not formally peer reviewed prior to publication, but still undergo a certain degree of moderation by experts in order to filter out non-scientific content. This practice represents a significant shift, as public dissemination was decoupled from a formalised editorial peer review process. Such practice results in increased visibility and combined rates of citation for articles that are deposited both in repositories like *arXiv* and traditional journal venues ( Davis & Fromerth, 2007; Moed, 2007).

The launch of *Open Journal Systems* ( kp.sfu.ca/ojs/; OJS) in 2001 offered a step towards bringing journals and peer review back to their community-led roots, by providing the technology to implement a range of potential peer review models within a low-cost open source platform. As of 2015, the OJS platform provided the technical infrastructure and editorial and peer review workflow management support to more than 10,000 journals ( Public Knowledge Project, 2016). Its exceptionally low cost was perhaps responsible for around half of these journals appearing in the developing world ( Edgar & Willinsky, 2010).

The past five to ten years have seen an accelerating wave of innovation in peer review, which we term “the revolution” phase ( Figure 2; note that this is a non-exhaustive overview of the peer review landscape). Initiatives such as the San Francisco Declaration on Research Assessment ( ascb.org/dora/; DORA), that called for systemic changes in the way that scientific research outputs are evaluated, and advances in Web-based technologies, are likely catalysts for such innovation. Born-digital journals, such as the *PLOS* series, introduced commenting on published papers, and Rapid Responses by *BMJ* has been highly successful in providing a platform for formalised comments ( bmj.com/rapid-responses). Such initiatives spurred developments in cross-publisher annotation platforms like *PubPeer* ( pubpeer.com/) and *PaperHive* ( paperhive.org/). Some journals, such as *F1000 Research* ( f1000research.com/) and *The Winnower* ( thewinnower.com/), rely exclusively on a model where peer review is conducted after the manuscripts are made publicly available. Other services, such as *Publons* ( publons.com/), enable reviewers to claim recognition for their activities as referees. Originally, *Academic Karma* ( academickarma.org/) offered a similar service to *Publons*, but has since adapted its model to facilitate peer review of preprints. Platforms such as *ScienceOpen* ( scienceopen.com/) provide a search engine combined with peer review across publishers on all documents, regardless of whether manuscripts have been previously reviewed. Each of these innovations has partial parallels to other social Web applications or platforms in terms of transparency, reputation, performance assessment, and community engagement. It remains to be seen whether these new models of evaluation will become more popular than traditional peer review, either singularly or in combination.

***1.1.4 Evidence from studies of peer review.*** Several empirical studies on peer review have been reported in the past few decades, mostly at the journal- or population-level. These studies typically use several different approaches to gather evidence on the functionality of peer review. Some, such as Bornmann & Daniel (2010b); Daniel (1993); Zuckerman & Merton (1971), used access to journal editorial archives to calculate acceptances, assess inter-reviewer agreement, and compare acceptance rates to various article, topic, and author features. Others interviewed or surveyed authors, reviewers, and editors to assess attitudes and behaviours, while others conducted randomized controlled trials to assess aspects of peer review bias ( Justice *et al.*, 1998; Overbeke, 1999). A systematic review of these studies concluded that evidence supporting the effectiveness of peer review training initiatives was inconclusive ( Galipeau *et al.*, 2015), and that major knowledge gaps existed in our application of peer review as a method to ensure high quality of scientific research outputs.

In spite of such studies, there appears to be a widening gulf between the rate of innovation and the availability of quantitative, empirical research regarding the utility and validity of modern peer review systems ( Squazzoni *et al.*, 2017a; Squazzoni *et al.*, 2017b). This should be deeply concerning given the significance that has been attached to peer review as a form of community moderation in scholarly research. Indeed, very few journals appear to be committed to objectively assess their effectiveness during peer review ( Lee & Moher, 2017). The consequence of this is that much remains unknown about the “black box” of peer review, as it is sometimes called ( Smith, 2006). The optimal designs for understanding and assessing the effectiveness of peer review, and therefore improving it, remain poorly understood, as the data required to do so are often not available ( Bruce *et al.*, 2016; Galipeau *et al.*, 2015). This also makes it very hard to measure and assess the quality, standard, and consistency of peer review not only between articles and journals, but also on a system-wide scale in the scholarly literature. Research into such aspects of peer review is quite time-consuming and intensive, particularly when investigating traits such as validity, and often criteria for assessing these are based on post-hoc measures such as citation frequency.

Despite the criticisms levied at the implementation of peer review, it remains clear that the ideal of it still plays a fundamental role in scholarly communication ( Goodman *et al.*, 1994; Mulligan *et al.*, 2013; Pierie *et al.*, 1996; Ware, 2008) and retains a high level of respect from the research community ( Bedeian, 2003; Gibson *et al.*, 2008; Greaves *et al.*, 2006; Mulligan *et al.*, 2013). One primary reason why peer review has persisted is that it remains a unique way of assigning credit to authors and differentiating research publications from other types of literature, including blogs, media articles, and books. This perception, combined with a general lack of awareness or appreciation of the historic evolution of peer review, research examining its potential flaws, and the conflation of the process with the ideology, has sustained its near-ubiquitous usage and continued proliferation in academia. There remains a widely-held perception that peer review is a singular and static process, and thus its wide acceptance as a social norm. It is difficult to move away from a process that has now become so deeply embedded within global research institutes. The consequence of this is that validation offered through peer review remains one of the essential pillars of trust in scholarly communication, irrespective of any potential flaws ( Haider & Åström, 2017).

In the following section, we summarize the ebb and flow of the debate around the various and complex aspects of conventional peer review. In particular, we highlight how innovative systems are attempting to resolve some of the major issues associated with traditional models, explore how new platforms could improve the process in the future, and consider what this means for the identity, role, and purpose of peer review within diverse research communities. The aim of this discussion is not to undermine any specific model of peer review in a quest for systemic upheaval, or to advocate any particular alternative model. Rather, we acknowledge that the idea of peer review is critical for research and advancing our knowledge, and as such we provide a foundation for future exploration and creativity in improving an essential component of scholarly communication.

### 1.2 The role and purpose of modern peer review

The systematic use of external peer review has become entwined with the core activities of scholarly communication. Without approval through peer review to assess importance, validity, and journal suitability, research articles do not become part of the body of scientific knowledge. While in the digital world the costs of dissemination are very low, the marginal cost of publishing articles is far from zero (e.g., due to time and management, hosting, marketing, and technical and ethical checks). The economic motivations for continuing to impose selectivity in a digital environment, and applying peer review as a mechanism for this, have received limited attention or questioning, and are often simply regarded as how things are done. Use of selectivity is now often attributed to quality control, but may be more about building the brand and the demand from specific publishers or venues. Proprietary reviewer databases that enable high selectivity are seen as a good business asset. In fact, the attribution is based on the false assumption that peer review requires careful selection of specific reviewers to assure a definitive level of adequate quality, termed the “Fallacy of Misplaced Focus” by Kelty *et al.* (2008).

In addition to being used to judge submitted material for acceptance at a journal, review comments provided to the authors serve to improve the work and the writing and analysis skills of the authors. This feedback can lead to improvements to the submitted work that are iterated between the authors, reviewers, and editor, until the work is either accepted or the editor decides that it cannot be made acceptable for their specific scientific journal. In other cases, it allows the authors to improve their work to prepare for a new submission to another venue. In both cases, a good (i.e., constructive) peer review should provide general feedback that allows authors to improve their skills and competency at preparing and presenting their research. In a sense, good peer review can serve as distributed mentorship.

In many cases, there is an attempt to link the goals of peer review processes with Mertonian norms ( Lee *et al.*, 2013; Merton, 1973) (i.e., universalism, communalism, disinterestedness, and organized scepticism) as a way of showing their relation to shared community values. The Mertonian norm of organized scepticism is the most obvious link, while the norm of disinterestedness can be linked to efforts to reduce systemic bias, and the norm of communalism to the expectation of contribution to peer review as part of community membership (i.e., duty). In contrast to the emphasis on supposedly shared social values, relatively little attention has been paid to the diversity of processes of peer review across journals, disciplines, and time (an early exception is Zuckerman & Merton (1971)). This is especially the case as the (scientific) scholarly community appears overall to have a strong investment in a “creation myth” that links the beginning of scholarly publishing—the founding of *The Philosophical Transactions of the Royal Society*—to the invention of peer review. The two are often regarded to be coupled by necessity, largely ignoring the complex and interwoven histories of peer review and publishing. This has consequences, as the individual identity of a scholar is strongly tied to specific forms of publication that are evaluated in particular ways ( Moore *et al.*, 2017). A scholar’s first research article, doctoral thesis, or first book are significant life events. Membership of a community, therefore, is validated by the peers who review this newly contributed work. Community investment in the idea that these processes have “always been followed” appears very strong, but ultimately remains a fallacy.

As mentioned above, there is an increasing quantity and quality of research that examines how publication processes, selection, and peer review evolved from the 17th to the early 20th century, and how this relates to broader social patterns ( Baldwin, 2017a; Baldwin, 2017b; Fyfe *et al.*, 2017; Moxham & Fyfe, 2017). However, much less research critically explores the diversity of selection of peer review processes in the mid- to late-20th century. Indeed, there seems to be a remarkable discrepancy between the historical work we do have ( Baldwin, 2017a; Gupta, 2016; Rennie, 2016; Shuttleworth & Charnley, 2016) and apparent community views that “we have always done it this way,” alongside what sometimes feels like a wilful effort to ignore the current diversity of practice. The result of this is an overall lack of evidence about the mechanics of peer review (e.g., time taken to review, conflict resolution, demographics of engaged parties, acceptance rates, quality of reviews, inherent biases, impact of referee training), both in terms of the traditional process and ongoing innovations, that obfuscates our understanding of the functionality and effectiveness of the present system ( Jefferson *et al.*, 2007). However, such a lack of evidence should not be misconstrued as evidence for the failure of these systems, but interpreted more as representing difficulties in empirically assessing the effectiveness of a diversity of practices in peer review.

Such a discrepancy between a dynamic history and remembered consistency could be a consequence of peer review processes being central to both scholarly identity as a whole and to the identity and boundaries of specific communities ( Moore *et al.*, 2017). Indeed, this story linking identity to peer review is taught to junior researchers as a community norm, often without the much-needed historical context. More work on how peer review, alongside other community practices, contributes to community building and sustainability would be valuable. Examining criticisms of conventional peer review and proposals for change through the lens of community formation and identity may be a productive avenue for future research.

### 1.3 Criticisms of the conventional peer review system

The debates surrounding the efficacy and implementation of peer review are becoming increasingly heated, and it is now not uncommon to hear claims that it is “broken” or “dysfunctional”. In spite of its clear relevance, widespread acceptance, and long-standing practice, the academic research community does not appear to have a clear or unified consensus on the operational functionality of peer review, and what its effects in a diverse modern research world are. One of the major consequences of this is that there remains a discrepancy between how peer review is regarded as a process and how it is actually performed. While peer review is still generally perceived as key to quality control for research, it has been argued that mistakes are becoming more frequent in the process ( Margalida & Colomer, 2016; Smith, 2006), and that peer review is not being applied as rigorously as generally perceived. As a result, it has become the target of widespread criticism, with a range of empirical studies investigating the reliability, credibility and fairness of the scholarly publishing and peer review process (e.g., ( Bruce *et al.*, 2016; Cole, 2000; Eckberg, 1991; Ghosh *et al.*, 2012; Jefferson *et al.*, 2002; Kostoff, 1995; Ross-Hellauer, 2017; Schroter *et al.*, 2006; Walker & Rocha da Silva, 2015)). In response to issues with quality in research articles, initiatives like the EQUATOR network ( equator-network.org) have been important to improve the reporting of research and its peer review according to standardised criteria. Another response to issues with scholarly publishing has been COPE, the Committee on Publication Ethics ( publicationethics.org), established in 1997 to address potential cases of abuse and misconduct during the publication process (specifically regarding author misconduct), and later created specific guidelines for peer review. Yet, the effectiveness of this initiative at a system-level remains unclear. A popular and widely-cited editorial in *The BMJ* made some quite serious allegations at peer review, stating that it is “slow, expensive, profligate of academic time, highly subjective, prone to bias, easily abused, poor at detecting gross defects, and almost useless at detecting fraud” ( Smith, 2006). In addition, beyond editorials, a substantial corpus of studies has now critically examined many of the various technical aspects of conventional journal article peer review (e.g., ( Armstrong, 1997; Bruce *et al.*, 2016; Jefferson *et al.*, 2007; Overbeke, 1999; Pöschl, 2012; Siler *et al.*, 2015a)), with overlapping and some times contrasting results.

Ultimately, the issue is that this uncertainty in standards and implementation can, at least in part, potentially lead to widespread failures in research quality and integrity ( Ioannidis, 2005; Jefferson *et al.*, 2002), and even the rise of formal retractions in extreme cases ( Steen *et al.*, 2013). Issues resulting from peer review failure range from simple subjective gate-keeping errors, often based on differences in opinion of the perceived impact of research, to failing to detect fraudulent or incorrect work, which then enters the scientific record and relies on post-publication evaluation (e.g., retraction) to correct ( Baxt *et al.*, 1998; Gøtzsche, 1989; Haug, 2015; Moore *et al.*, 2017; Pocock *et al.*, 1987; Schroter *et al.*, 2004; Smith, 2006). A final issue regards peer review by and for non-native English speaking authors, which can lead to cases of linguistic inequality and language-oriented research segregation, in a world where research is increasingly becoming more globally competitive ( Salager-Meyer, 2008, Salager-Meyer, 2014). Such criticisms should be a cause for concern given that traditional peer review is still viewed by some, almost by concession, as a gold standard and requirement for the publication of research results ( Mayden, 2012). All of this suggests that, while the concept of peer review remains logical and required, it is the practical implementation of it that demands further attention.

***1.3.1 Peer review needs to be peer reviewed.*** Attempts to reproduce how peer review selects what is worthy of publication demonstrate that the process is generally adequate for detecting reliable research, but often fails to recognize the research that has the greatest impact ( Mahoney, 1977; Moore *et al.*, 2017; Siler *et al.*, 2015b). Many critics now view traditional peer review as sub-optimal and detrimental to research because it causes publication delays, with repercussions on the dissemination of novel research ( Armstrong, 1997; Bornmann & Daniel, 2010a; Brembs, 2015; Eisen, 2011; Jubb, 2016; Vines, 2015b). Reviewer fatigue and redundancy when articles go through multiple rounds of peer review at different journal venues ( Breuning *et al.*, 2015; Fox *et al.*, 2017; Jubb, 2016; Moore *et al.*, 2017) are just some of the major criticisms levied at the technical implementation of peer review. In addition, some view many common forms of peer review as flawed because they operate within a closed and opaque system. This makes it impossible to trace the discussions that led to (sometimes substantial) revisions to the original research ( Bedeian, 2003), the decision process leading to the final publication, or whether peer review even took place. By operating as a closed system, it protects the status quo and suppresses research viewed as radical, innovative, or contrary to the theoretical or established perspectives of referees ( Alvesson & Sandberg, 2014; Benda & Engels, 2011; Horrobin, 1990; Mahoney, 1977; Merton, 1968; Siler *et al.*, 2015a; Siler & Strang, 2017), even though it is precisely these factors that underpin and advance research. As a consequence, questions arise as to the competency, effectiveness, and integrity, as well as participatory elements, of traditional peer review, such as: who are the gatekeepers and how are the gates constructed; what is the balance between author-reviewer-editor tensions and how are these power relations and conflicts resolved; what are the inherent biases associated with this; does this enable a fair or structurally inclined system of peer review to exist; and what are the repercussions for this on our knowledge generation and communication systems?

## 2 The traits and trends affecting modern peer review

Over time, three principal forms of journal peer review have evolved: single blind, double blind, and open ( Table 1). Of these, single blind, where reviewers are anonymous but authors are not, is the most widely-used in most disciplines because the process is considered to be more impartial, and comparably less onerous and less expensive to operate than the alternatives. Double blind peer review, where both authors and reviewers are reciprocally anonymous, requires considerable effort to remove all traces of the author’s identity from the manuscript under review ( Blank, 1991). For a detailed comparison of double versus single blind review, Snodgrass (2007) provides an excellent summary. The advent of “open peer review” introduced substantial additional complexity into the discussion ( Ross-Hellauer, 2017).

**Table 1.** Types of reciprocal identification or anonymity in the peer review process.

		*Author Identity*	
		Hidden	Known
***Reviewer*** ***Identity***	**Hidden**	Double blind	Single blind
	**Known**	–	Open

The recent diversification of peer review is intrinsically coupled with wider developments in scholarly publishing. When it comes to the gate-keeping function of peer review, innovation is noticeable in some digital-only, or “born open,” journals, such as *PLOS ONE* and *PeerJ*. These explicitly request referees to ignore any notion of novelty, significance, or impact, before it becomes accessible to the research community. Instead, reviewers are asked to focus on whether the research was conducted properly and that the conclusions are based on the presented results. This arguably more objective method has met some resistance, even receiving the somewhat derogatory term “peer review lite” from some corners of the scholarly publishing industry ( Pinfield, 2016). Such a sentiment can be viewed as a hangover from the commercial age of non-digital publishing, and now seems superfluous and discordant with any modern Web-based model of scholarly communication. Indeed, when *PLOS ONE* started publishing in 2006, it initiated the phenomenon of open access “mega journals”, which had distinct publishing criteria to traditional journals (i.e., broad scope, large size, objective peer review), and which have since become incredibly successful ventures ( Wakeling *et al.*, 2016). Some even view the desire for emphasis on novelty in publishing to have counter-productive effects on scientific progress and the organization of scientific communities ( Cohen, 2017), and journals based on the model of *PLOS ONE* represent a solution to this. The relative timing of peer review to publication is a further major innovation, with journals such as *F1000 Research* publishing prior to any formal peer review, with the process occurring continuously and articles updated iteratively. Some of the advantages and disadvantages of these different variations of peer review are explored in Table 2.

**Table 2.** Advantages and disadvantages of the different approaches to peer review. Note that combinations of these approaches can co-exist. NPRC: Neuroscience Peer Review Consortium.

Type	Description	Pros/Benefits	Cons/Risks	Examples
Pre-peer review commenting	Informal commenting and discussion on a publicly available pre-publication manuscript draft (i.e., preprints)	Rapid, transparent, public, relatively low cost (free for authors), open commenting	Variable uptake, fear of scooping, fear of journal rejection, fear of premature communication, no editorial control	bioRxiv, OSF Preprints, PeerJ Preprints, Figshare, Zenodo, Preprints.org
Pre-publication (closed)	Formal and editorially-invited evaluation of a piece of research by selected experts in the relevant field	Editorial moderation, provides at least some form of quality control for all published work	Mostly non-transparent, difficult to evaluate, potentially biased, secretive and exclusive, unclear who “owns” reviews	Nature, Science, New England Journal of Medicine, Cell, The Lancet
Post-publication	Formal and optionally-invited evaluation of research by selected experts in the relevant field, subsequent to publication	Rapid publication of research, public, transparent, can be editorially-moderated, continuous	Filtering of “bad research” occurs after publication, relatively low uptake	F1000 Research, ScienceOpen, RIO, The Winnower, Publons
Post-publication commenting	Informal discussion of published research, independent of any formal peer review that may have already occurred	Can be performed on third-party platforms, anyone can contribute, public	Comments can be rude or of low quality, comments across multiple platforms lack inter-operability, low visibility, low uptake	PubMed Commons, PeerJ, PLOS, BMJ
Collaborative	A combination of referees, editors and external readers participate in the assessment of scientific manuscripts through interactive comments, often to reach a consensus decision, and a single set of revisions	Iterative, transparent, editors sign reports, can be integrated with formal process, deters low quality submissions	Can be additionally time-consuming, discussion quality variable, peer pressure and influence can tilt the balance	eLife, Frontiers series, Copernicus journals, BMJ Open Science
Portable	Authors can take referee reports to multiple consecutive venues, often administered by a third-party service	Reduces redundancy or duplication, saves time	Low uptake by authors, low acceptance by journals, high cost	BioMed Central journals, NPRC, Rubriq, Peerage of Science, MECA
Recommendation services	Post-publication evaluation and recommendation of significant articles, often through a peer- nominated consortium	Crowd-sourced literature discovery, time saving, “prestige” factor when inside a consortium	Paid services (subscription only), time consuming on recommender side, exclusive	F1000 Prime, CiteULike
Decoupled post-publication (annotation services)	Comments or highlights added directly to highlighted sections of the work. Added notes can be private or public	Rapid, crowd-sourced and collaborative, cross-publisher, low threshold for entry	Non-interoperable, multiple venues, effort duplication, relatively unused, genuine critiques reserved	PubPeer, Hypothesis, PaperHive, PeerLibrary

### 2.1 The development of open peer review

New suggestions to modify peer review vary, between fairly incremental small-scale changes, to those that encompass an almost total and radical transformation of the present system. A core question is how to transform traditional peer review into a process that is aligned with the latest advances in what is now widely termed “open science”. This is tied to broader developments in how we as a society communicate, thanks to the inherent capacity that the Web provides for open, collaborative, and social communication. Many of the suggestions and new models for opening peer review up are geared towards increasing different levels of transparency, and ultimately the reliability, efficiency, and accountability of the publishing process. These traits are desired by all actors in the system, and increasing transparency moves peer review towards a more open model.

Novel ideas about “Open Peer Review” (OPR) systems are rapidly emerging, and innovation has been accelerating over the last several years ( Figure 2; Table 3). The advent of OPR is complex, as the term can refer to multiple different parts of the process and is often used inter-changeably or conflated without appropriate prior definition. Currently, there is no formally established definition of OPR that is accepted by the scholarly research and publishing community ( Ford, 2013). The most simple definitions by McCormack (2009) and Mulligan *et al.* (2008) presented OPR as a process that does not attempt “to mask the identity of authors or reviewers” ( McCormack, 2009, p.63), thereby explicitly referring to open in terms of personal identification or anonymity. Ware (2011, p.25) expanded on reviewer disclosure practices: “Open peer review can mean the opposite of double blind, in which authors’ and reviewers’ identities are both known to each other (and sometimes publicly disclosed), but discussion is complicated by the fact that it is also used to describe other approaches such as where the reviewers remain anonymous but their reports are published.” Other authors define OPR distinctly, for example by including the publication of all dialogue during the process ( Shotton, 2012), or running it as a publicly participative commentary ( Greaves *et al.*, 2006).

**Table 3.** Pros and cons of different approaches to anonymity in peer review.

Approach	Description	Pros/Benefits	Cons/Risks	Examples
Single blind peer review	Referees are not revealed to the authors, but referees are aware of author identities	Allows reviewers to view full context of an author’s other work, detection of COIs, more efficient	Prone to bias, authors not protected, exclusive, non-verifiable, referees can often be identified anyway	Most biomedical and physics journals, PLOS ONE, Science
Double blind peer review	Authors and the referees are reciprocally anonymous	Increased author diversity in published literature, protects authors and reviewers from bias, more objective	Still prone to abuse and bias, secretive, exclusive, non- verifiable, referees can often be identified anyway, time consuming	Nature, most social sciences journals
Triple-blind peer review	Authors and their affiliations are reciprocally anonymous to handling editors and reviewers	Eliminates geographical, institutional, personal and gender biases, work evaluated based on merit	Incompatible with pre- prints, low-uptake, non- verifiable, secretive	Science Matters
Private, open peer review	Referee names are revealed to the authors pre-publication, if the referees agree, either through an opt-in or opt-out mechanism	Protects referees, no fear of reprisal for critical reviews	Increases decline to review rates, non- verifiable	PLOS Medicine, Learned Publishing
Unattributed peer review	If referees agree, their reports are made public but anonymous when the work is published	Reports publicized for context and re-use	Prone to abuse and bias similar to double blind process, non-verifiable	EMBO Journal
Optional open peer review	As single blind peer review, except that the referees are given the option to make their review and their name public	Increased transparency	Gives an unclear pictures of the review process if not all reviews are made public	PeerJ, Nature Communications
Pre-publication open peer review	Referees are identified to authors pre-publication, and if the article is published, the full peer review history together with the names of the associated referees is made public	Transparency, increased integrity of reviews	Fear: referees may decline to review, or be unwilling to come across too critically or positively	The medical BMC-series journals, The BMJ
Post-publication open peer review	The referee reports and the names of the referees are always made public regardless of the outcome of their review	Fast publication, transparent process	Fear: referees may decline to review, or be unwilling to come across too critically or positively	F1000Research, ScienceOpen, PubPub, Publons
Peer review by endorsement (PRE)	Pre-arranged and invited, with referees providing a “stamp of approval” on publications	Transparent, cost- effective, rapid, accountable	Low uptake, prone to selection bias, not viewed as credible	RIO Journal

However, the context of this transparency and the implications of different modes of transparency at different stages of the review process are both very rarely explored. Progress towards achieving transparency has been variable but generally slow across the publishing system. Engagement with experimental open models is still far from common, in part perhaps due to a lack of rigorous evaluation and empirical demonstration that they are more effective processes. A consequence of this is the entrenchment of the ubiquitously practiced and much more favored traditional model (which, as noted above, is also diverse). However, as history shows, such a process is non-traditional but nonetheless currently held in high regard. Practices such as self-publishing and predatory or deceptive publishing cast a shadow of doubt on the validity of research posted openly online that follow these models, including those with traditional scholarly imprints ( Fitzpatrick, 2011a; Tennant *et al.*, 2016). The inertia hindering widespread adoption of new models of peer review can be ascribed to what is often termed “cultural inertia”, and affects many aspects of scholarly research. Cultural inertia, the tendency of communities to cling to a traditional trajectory, is shaped by a complex ecosystem of individuals and groups. These often have highly polarized motivations (i.e., capitalistic commercialism versus knowledge generation versus careerism versus output measurement), and an academic hierarchy that imposes a power dynamic that can suppress innovative practices ( Burris, 2004; Magee & Galinsky, 2008).

How and where we inject transparency has implications for the magnitude of transformation required and, therefore, the general concept of OPR is highly heterogeneous in meaning, scope, and consequences. A recent survey by *OpenAIRE* found 122 different definitions of OPR in use, exemplifying the extent of this issue. This diversity was distilled into a single proposed definition comprising seven different traits of OPR: participation, identity, reports, interaction, platforms, pre-review manuscripts, and final-version commenting ( Ross-Hellauer, 2017). The various parts of the “revolutionary” phase of peer review undoubtedly have different combinations of these OPR traits, and it remains a very heterogeneous landscape. Table 3 provides an overview of the advantages and disadvantages of the different approaches to anonymity and openness in peer review.

The ongoing discussions and innovations around peer review (and OPR) can be sorted into four main categories, which are examined in more detail below. Each of these feed into the wider core issues in peer review of incentivizing engagement, providing appropriate recognition and certification, and quality control and moderation:

1. How can referees receive credit or recognition for their work, and what form should this take;
2. Should referee reports be published alongside manuscripts;
3. Should referees remain anonymous or have their identities disclosed;
4. Should peer review occur prior or subsequent to the publication process (i.e., publish then filter).

### 2.2 Giving credit to peer reviewers

A vast majority of researchers see peer review as an integral and fundamental part of their work Mulligan *et al.* (2013). They often consider peer review to be part of an altruistic cultural duty or a quid pro quo service, closely associated with the identity of being part of their research community. To be invited to review a research article can be perceived as a great honor, especially for junior researchers, due to the recognition of expertise—i.e., the attainment of the level of a peer. However, the current system is facing new challenges as the number of published papers continues to increase rapidly ( Albert *et al.*, 2016), with more than one million articles published in peer reviewed, English-language journals every year ( Larsen & Von Ins, 2010). Some estimates are even as high as 2–2.5 million per year ( Plume & van Weijen, 2014), and this number is expected to double approximately every nine years at current rates ( Bornmann & Mutz, 2015). Several potential solutions exist to make sure that the review process does not cause a bottleneck in the current system:

- Increase the total pool of potential referees,
- Editorial staff more thoroughly vet submissions prior to sending for review,
- Increase acceptance rates to avoid review duplication,
- Impose a production cap on authors,
- Decrease the number of referees per paper, and/or
- Decrease the time spent on peer review.

Of these, the latter two can both potentially reduce the quality of peer review and therefore affect the overall quality of published research. Paradoxically, while the Web empowers us to communicate information virtually instantaneously, the turn around time for peer reviewed publications remains quite long by comparison. One potential solution is to encourage referees by providing additional recognition and credit for their work. The present lack of bona fide incentives for referees is perhaps one of the main factors responsible for indifference to editorial outcomes, which ultimately leads to the increased proliferation of low quality research ( D’Andrea & O’Dwyer, 2017; Jefferson *et al.*, 2007; Wang *et al.*, 2016).

***2.2.1 Traditional methods of recognition.*** One current way to recognize peer reviewers is to thank anonymous referees in the Acknowledgement sections of published papers. In these cases, the referees will not receive any public recognition for their work, unless they explicitly agree to sign their reviews. Generally, journals do not provide any remuneration or compensation for these services. Notable exceptions are the UK-based publisher *Veruscript* ( veruscript.com/about/who-we-are) and *Collabra* ( collabra.org/about/our-model), published by University of California Press, as well as most statistical referees ( Barbour, 2017). Other journals provide reward incentives to reviewers, such as free subscriptions or discounts on author-facing open access fees. Another common form of acknowledgement is a private thank you note from the journal or editor, which usually takes the form of an automated email upon completion of the review. In addition, journals often list and thank all reviewers in a special issue or on their website once a year, thus providing another way to recognise reviewers. Some journals even offer annual prizes to reward exceptional referee activities (e.g., the Journal of Clinical Epidemiology; www.jclinepi.com/article/S0895-4356(16)30707-7/fulltext). Another idea that journals and publishers have tried implementing is to list the best reviewers for their journal (e.g., by Vines (2015a) for *Molecular Ecology*), or, on the basis of a suggestion by Pullum (1984), naming referees who recommend acceptance in the article colophon (a single blind version of this recommendation was adopted by *Digital Medievalist* from 2005–2016; see Wikipedia contributors, 2017, and bit.ly/DigitalMedievalistArchive for examples preserved in the Internet Archive). *Digital Medievalist* stopped using this model and removed the colophon as part of its move to the *Open Library of Humanities*; cf. journal.digitalmedievalist.org). As such, authors can then integrate this into their scholarly profiles in order to differentiate themselves from other researchers or referees. Currently, peer review is poorly acknowledged by practically all research assessment bodies, institutions, granting agencies, as well as publishers, in the process of professional advancement or evaluation. Instead, it is viewed as expected or normal behaviour for all researchers to contribute in some form to peer review.

***2.2.2 Increasing demand for recognition.*** These traditional approaches of credit fall short of any sort of systematic feedback or recognition, such as that granted through publications. A change here is clearly required for the wealth of currently unrewarded time and effort given to peer review by academics. A recent survey of nearly 3,000 peer reviewers by the large publisher *Wiley* showed that feedback and acknowledgement for work as referees are valued far above either cash reimbursements or payment in kind ( Warne, 2016) (although Mulligan *et al.* (2013) found that referees would prefer either compensation by way of free subscriptions, or the waiver of colour or other publication charges). *Wiley’s* survey reports that 80% of researchers agree that there is insufficient recognition for peer review as a valuable research activity and that researchers would actually commit more time to peer review if it became a formally recognized activity for assessments, funding opportunities, and promotion ( Warne, 2016). While this may be true, it is important to note that commercial publishers have a vested interest in retaining the current, freely provided service of peer review, since this is what provides their journals the main stamp of legitimacy and quality (“added value”) as society-led journals. Therefore, one of the root causes for the lack of appropriate recognition and incentivization is publishers with have strong motivations to find non-monetary forms of reviewer recognition. Indeed, the business model of almost every scholarly publisher is predicated on free work by peer reviewers, and it is unlikely that the present system would function financially with market-rate reimbursement for reviewers. Other research shows a similar picture, with approximately 70% of respondents to a small survey done by Nicholson & Alperin (2016) indicating that they would list peer review as a professional service on their curriculum vitae. 27% of respondents mentioned formal recognition in assessment as a factor that would motivate them to participate in public peer review. These numbers indicate that the lack of credit referees receive for peer review is likely a strong contributing factor to the perceived stagnation of traditional models. Furthermore, acceptance rates are lower in humanities and social sciences, and higher in physical sciences and engineering journals ( Ware, 2008), as well as differences based on relative referee seniority ( Casnici *et al.*, 2017). This means there are distinct disciplinary variations in the number of reviews performed by a researcher relative to their publications, and suggests that there is scope for using this to either provide different incentive structures or to increase acceptance rates and therefore decrease referee fatigue ( Fox *et al.*, 2017; Lyman, 2013).

***2.2.3 Progress in crediting peer review.*** Any acknowledgement model to credit reviewers also raises the obvious question of how to facilitate this model within an anonymous peer review system. By incentivizing peer review, much of its potential burden can be alleviated by widening the potential referee pool concomitant with the growth in review requests. This can also help to diversify the process and inject transparency into peer review, a solution that is especially appealing when considering that it is often a small minority of researchers who perform the vast majority of peer reviews ( Fox *et al.*, 2017; Gropp *et al.*, 2017); for example, in biomedical research, only 20 percent of researchers perform 70–95 percent of the reviews ( Kovanis *et al.*, 2016). In 2014, a working group on peer review services (CASRAI) was established to “develop recommendations for data fields, descriptors, persistence, resolution, and citation, and describe options for linking peer-review activities with a person identifier such as *ORCID*” ( Paglione & Lawrence, 2015). The idea here is that by being able to standardize the description of peer review activities, it becomes easier to attribute, and therefore recognize and reward them.

The *Publons* platform provides a semi-automated mechanism to formally recognize the role of editors and referees who can receive due credit for their work as referees, both pre- and post-publication. Researchers can also choose if they want to publish their full reports depending on publisher and journal policies. *Publons* also provides a ranking for the quality of the reviewed research article, and users can endorse, follow, and recommend reviews. Other platforms, such as *F1000 Research* and *ScienceOpen*, link post-publication peer review activities with *CrossRef* DOIs and open licenses to make them more citable, essentially treating them equivalent to a normal open access research paper. *ORCID* (Open Researcher and Contributor ID) provides a stable means of integrating these platforms with persistent researcher identifiers in order to receive due credit for reviews. *ORCID* is rapidly becoming part of the critical infrastructure for open OPR, and greater shifts towards open scholarship ( Dappert *et al.*, 2017). Exposing peer reviews through these platforms links accountability to receiving credit. Therefore, they offer possible solutions to the dual issues of rigor and reward, while potentially ameliorating the growing threat of reviewer fatigue due to increasing demands on researchers external to the peer review system ( Fox *et al.*, 2017; Kovanis *et al.*, 2016).

Whether such initiatives will be successful remains to be seen However, *Publons* was recently acquired by *Clarivate Analytics*, suggesting that the process could become commercialized as this domain rapidly evolves ( Van Noorden, 2017). In spite of this, the outcome is most likely to be dependent on whether funding agencies and those in charge of tenure, hiring, and promotion will use peer review activities to help evaluate candidates. This is likely dependent on whether research communities themselves choose to embrace any such crediting or accounting systems for peer review.

### 2.3 Publishing peer review reports

The rationale behind publishing referee reports lies in providing increased context and transparency to the peer review process, and can occur irrespective of whether or not the reviewers reveal their identities. Often, valuable insights are shared in reviews that would otherwise remain hidden if not published. By publishing reports, peer review has the potential to become a supportive and collaborative process that is viewed more as an ongoing dialogue between groups of scientists to progressively assess the quality of research. Furthermore, the reviews themselves are opened up for analysis and inspection, including how authors respond to reviews, adding an additional layer of quality control and a means for accountability and verification. There are additional educational benefits to publishing peer reviews, such as training purposes or for journal clubs. Given the inconclusive evidence regarding the training of referees ( Galipeau *et al.*, 2015; Jefferson *et al.*, 2007), such practices might be further useful in highlighting our knowledge and skills gaps. At the present, some publisher policies are extremely vague about the re-use rights and ownership of peer review reports ( Schiermeier, 2017). The *Peer Review Evaluation (PRE)* service ( www.pre-val.org) was designed to breathe some transparency into peer review, and provide information about the peer review itself without exposing the reports (e.g., mode of peer review, number of referees, rounds of review). While it describes itself as a service to identify fraud and maintain the integrity of peer review, it remains unclear whether it has achieved these objectives in light of the ongoing criticisms of the conventional process.

In a study of two journals, one where reports were not published and another where they were, Bornmann *et al.* (2012) found that publicized comments were much longer by comparison. Furthermore, there was an increased chance that they would result in a constructive dialogue between the author, reviewers, and wider community, and might therefore be better for improving the content of a manuscript. On the other hand, unpublished reviews tended to have more of a selective function to determine whether a manuscript is appropriate for a particular journal (i.e., focusing on the editorial process). Therefore, depending on the journal, different types of peer review could be better suited to perform different functions, and therefore optimized in that direction. Transparency of the peer review process can also be used as an indicator for peer review quality, thereby potentially enabling the tool to predict quality in new journals in which the peer review model is known, if desired ( Godlee, 2002; Morrison, 2006; Wicherts, 2016). Journals with higher transparency ratings were less likely to accept flawed papers and showed a higher impact as measured by Google Scholar’s h5-index ( Wicherts, 2016).

Assessments of research articles can never be evidence-based without the verification enabled by publication of referee reports. However, they are still almost ubiquitously regarded as having an authoritative, and uniform, stamp of quality. The issue here is that the attainment of peer reviewed status will always be based on an undefined, and only ever relative, quality threshold due to the opacity of the process. This is in itself quite an unscientific practice, and instead, researchers rely almost entirely on heuristics and trust for a concealed process and the intrinsic reputation of the journal, rather than anything legitimate. This can ultimately result in what is termed the “Fallacy of Misplaced Finality”, described by Kelty *et al.* (2008), as the assumption that research has a single, final form, to which everyone applies different criteria of quality.

Publishing peer review reports appears to have little or no impact on the overall process but may encourage more civility from referees. In a small survey, Nicholson & Alperin (2016) found that approximately 75% of survey respondents (n=79) perceived that public peer review would change the tone or content of the reviews, and 80% of responses indicated that performing peer reviews that would be eventually be publicized would not require a significantly higher amount of work. However, the responses also indicated that incentives are needed for referees to engage in this form of peer review. This includes recognition by performance review or tenure committees (27%), peers publishing their reviews (26%), being paid in some way such as with an honorarium or waived APC (24%), and getting positive feedback on reviews from journal editors (16%). Only 3% (one response) indicated that nothing could motivate them to participate in an open peer review of this kind. Leek *et al.* (2011) showed that when referees’ comments were made public, significantly more cooperative interactions were formed, while the risk of incorrect comments decreased, suggesting that prior knowledge of publication encourages referees to be more constructive and careful with their reviews. Moreover, referees and authors who participated in cooperative interactions had a reviewing accuracy rate that was 11% higher. On the other hand, the possibility of publishing the reviews online has also been associated with a high decline rate among potential peer reviewers, and an increase in the amount of time taken to write a review, but with a variable effect on review quality ( Almquist *et al.*, 2017; van Rooyen *et al.*, 2010). This suggests that the barriers to publishing review reports are inherently social, rather than technical.

When *BioMed Central* launched in 2000, it quickly recognized the value in including both the reviewers’ names and the peer review history (pre-publication) alongside published manuscripts in their medical journals in order to increase the quality and value of the process. Since then, further reflections on OPR ( Godlee, 2002) led to the adoption of a variety of new models. For example, the *Frontiers* series now publishes all referee names alongside articles, *EMBO* journals publish a review process file with the articles, with referees remaining anonymous but editors being named, and *PLOS* added public commenting features to articles they published in 2009. More recently launched journals such as *PeerJ* have a system where both the reviews and the names of the referees can optionally be made public, and journals such as *Nature Communications* and the *European Journal of Neuroscience* have also started to adopt this method.

Unresolved issues with posting review reports include whether or not it should be conducted for ultimately unpublished manuscripts, and the impact of author identification or anonymity alongside their reports. Furthermore, the actual readership and usage of published reports remains ambiguous in a world where researchers are typically already inundated with published articles to read. The benefits of publicizing reports might not be seen until further down the line from the initial publication and, therefore, their immediate value might be difficult to convey and measure in current research environments. Finally, different populations of reviewers with different cultural norms and identities will undoubtedly have varying perspectives on this issue, and it is unlikely that any single policy or solution to posting referee reports will ever be widely adopted. Further investigation of the link between making reviews public and the impact this has on their quality would be a fruitful area of research to potentially encourage increased adoption of this practice.

### 2.4 Eponymous versus anonymous peer review

**Figure 3.**
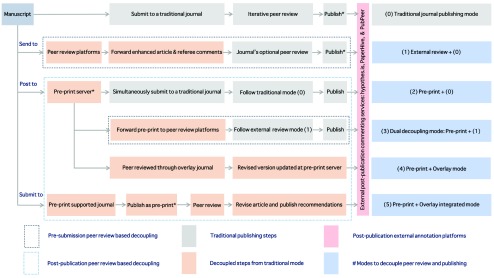
Traditional versus different decoupled peer review models: Peer review under decoupled model either happens pre-submission or post-publication. The dotted border lines in the figure highlight this element, with boxes colored in orange representing decoupled steps from the traditional publishing model (0) and the ones colored gray depicting the traditional publishing model itself. Pre-submission peer review based decoupling (1) offers a route to enhance a manuscript before submitting it to a traditional journal; post-publication peer review based decoupling follows preprint first mode through four different ways (2, 3, 4, and 5) for revision and acceptance. Dual-decoupling (3) is when a manuscript initially posted as a preprint (first decoupling) is sent for external peer review (second decoupling) before its formal submission to a traditional journal. The asterisks in the figure indicate when the manuscript first enters the public view irrespective of its peer review status.

There are different levels of bi-directional anonymity throughout the peer review process, including whether or not the referees know who the authors are but not vice versa (single blind; the most common ( Ware, 2008)), or whether both parties remain anonymous to each other (double blind) ( Table 1). Double blind review is based on the idea that peer evaluations should be impartial and based on the research, not ad hominem, but there has been considerable discussion over whether reviewer identities should remain anonymous (e.g., Baggs *et al.* (2008); Pontille & Torny (2014); Snodgrass (2007)) ( Figure 3). Models such as triple-blind peer review even go a step further, where authors and their affiliations are reciprocally anonymous to the handling editor and the reviewers. This attempts to nullify the effects of one’s scientific reputation, institution, or location on the peer review process, and is employed at the open access journal *Science Matters* ( sciencematters.io), launched in early 2016.

While there is much potential value in anonymity, the corollary is also problematic in that anonymity can lead to reviewers being more aggressive, biased, negligent, orthodox, entitled, and politicized in their language and evaluation, as they have no fear of negative consequences for their actions other than from the editor. ( Lee *et al.*, 2013; Weicher, 2008). In theory, anonymous reviewers are protected from potential backlashes for expressing themselves fully and therefore are more likely to be more honest in their assessments. Some evidence suggests that single-blind peer review has a detrimental impact on new authors, and strengthens the harmful effects of ingroup-outgroup behaviours ( Seeber & Bacchelli, 2017). Furthermore, by protecting the referees’ identities, journals lose an aspect of the prestige, quality, and validation in the review process, leaving researchers to guess or assume this important aspect post-publication. The transparency associated with signed peer review aims to avoid competition and conflicts of interest that can potentially arise for any number of financial and non-financial reasons, as well as due to the fact that referees are often the closest competitors to the authors, as they will naturally tend to be the most competent to assess the research ( Campanario, 1998a; Campanario, 1998b). There is additional evidence to suggest that double blind review can increase the acceptance rate of women-authored articles in the published literature ( Darling, 2015).

On the other hand, eponymous peer review has the potential to inject responsibility into the system by encouraging increased civility, accountability, declaration of biases and conflicts of interest, and more thoughtful reviews ( Boldt, 2011; Cope & Kalantzis, 2009; Fitzpatrick, 2010; Janowicz & Hitzler, 2012; Lipworth *et al.*, 2011; Mulligan *et al.*, 2013). Identification also helps to extend the process to become more of an ongoing, community-driven dialogue rather than a singular, static event ( Bornmann *et al.*, 2012; Maharg & Duncan, 2007). However, there is scope for the peer review to become less critical, skewed, and biased by community selectivity. If the anonymity of the reviewers is removed while maintaining author anonymity at any time during peer review, a skew and extreme accountability is imposed upon the reviewers, while authors remain relatively protected from any potential prejudices against them. However, such transparency provides, in theory, a mode of validation and should mitigate corruption as any association between authors and reviewers would be exposed. Yet, this approach has a clear disadvantage, in that accountability becomes extremely one-sided. Another possible result of this is that reviewers could be stricter in their appraisals within an already conservative environment, and thereby further prevent the publication of research. As such, we can see that strong, but often conflicting arguments and attitudes exist for both sides of the anonymity debate (see e.g., Prechelt *et al.* (2017); Seeber & Bacchelli (2017)), and are deeply linked to critical discussions about power dynamics in peer review ( Lipworth & Kerridge, 2011).

***2.4.1 Reviewing the evidence.*** Reviewer anonymity can be difficult to protect, as there are ways in which identities can be revealed, albeit non-maliciously. For example, through language and phrasing, prior knowledge of the research and a specific angle being taken, previous presentation at a conference, or even simple Web-based searches. Baggs *et al.* (2008) investigated the beliefs and preferences of reviewers about blinding. Their results showed double blinding was preferred by 94% of reviewers, although some identified advantages to an un-blinded process. When author names were blinded, 62% of reviewers could not identify the authors, while 17% could identify authors ≤10% of the time. Walsh *et al.* (2000) conducted a survey in which 76% of reviewers agreed to sign their reviews. In this case, signed reviews were of higher quality, were more courteous, and took longer to complete than unsigned reviews. Reviewers who signed were also more likely to recommend publication. In one study from the reviewers’ perspectives, Snell & Spencer (2005) found that they would be willing to sign their reviews and felt that the process should be transparent. Yet, a similar study by Melero & Lopez-Santovena (2001) found that 75% of surveyed respondents were in favor of reviewer anonymity, while only 17% were against it.

A randomized trial showed that blinding reviewers to the identity of authors improved the quality of the reviews ( McNutt *et al.*, 1990). This trial was repeated on a larger scale by Justice *et al.* (1998) and Van Rooyen *et al.* (1999), with neither study finding that blinding reviewers improved the quality of reviews. These studies also showed that blinding is difficult in practice, as many manuscripts include clues on authorship. Jadad *et al.* (1996) analyzed the quality of reports of randomized clinical trials and concluded that blind assessments produced significantly lower and more consistent scores than open assessments. The majority of additional evidence suggests that anonymity has little impact on the quality or speed of the review or of acceptance rates ( Isenberg *et al.*, 2009; Justice *et al.*, 1998; van Rooyen *et al.*, 1998), but revealing the identity of reviewers may lower the likelihood that someone will accept an invitation to review ( Van Rooyen *et al.*, 1999). Revealing the identity of the reviewer to a co-reviewer also has a small, editorially insignificant, but statistically significant beneficial effect on the quality of the review ( van Rooyen *et al.*, 1998). Authors who are aware of the identity of their reviewers may also be less upset by hostile and discourteous comments ( McNutt *et al.*, 1990). Other research found that signed reviews were more polite in tone, of higher quality, and more likely to ultimately recommend acceptance ( Walsh *et al.*, 2000). As such, the research into the effectiveness and impact of blinding, including the success rates of attempts of reviewers and authors to deanonymize each other, remains largely inconclusive (e.g., Blank (1991); Godlee *et al.* (1998); Goues *et al.* (2017); Okike *et al.* (2016); van Rooyen *et al.* (1998)).

***2.4.2 The dark side of identification.*** This debate of signed versus unsigned reviews, independently of whether reports are ultimately published, is not to be taken lightly. Early career researchers in particular are some of the most conservative in this area as they may be afraid that by signing overly critical reviews (i.e., those which investigate the research more thoroughly), they will become targets for retaliatory backlashes from more senior researchers ( Rodríguez-Bravo *et al.*, 2017). In this case, the justification for reviewer anonymity is to protect junior researchers, as well as other marginalized demographics, from bad behavior. Furthermore, author anonymity could potentially save junior authors from public humiliation from more established members of the research community, should they make errors in their evaluations. These potential issues are at least a part of the cause towards a general attitude of conservatism and a prominent resistance factor from the research community towards OPR (e.g., Darling (2015); Godlee *et al.* (1998); McCormack (2009); Pontille & Torny (2014); Snodgrass (2007); van Rooyen *et al.* (1998)). However, it is not immediately clear how this widely-exclaimed, but poorly documented, potential abuse of signed-reviews is any different from what would occur in a closed system anyway, as anonymity provides a potential mechanism for referee abuse. Indeed, the tone of discussions on platforms where anonymity or pseudonymity is allowed, such as *Reddit* or *PubPeer*, is generally problematic, with the latter even being referred to as facilitating “vigilante science” ( Blatt, 2015). The fear that most backlashes would be external to the peer review itself, and indeed occur in private, is probably the main reason why such abuse has not been widely documented. However, it can also be argued that by reviewing with the prior knowledge of open identification, such backlashes are prevented, since researchers do not want to tarnish their reputations in a public forum. Under these circumstances, openness becomes a means to hold both referees and authors accountable for their public discourse, as well as making the editors’ decisions on referee and publishing choice public. Either way, there is little documented evidence that such retaliations actually occur either commonly or systematically. If they did, then publishers that employ this model, such as *Frontiers* or *BioMed Central*, would be under serious question, instead of thriving as they are.

In an ideal world, we would expect that strong, honest, and constructive feedback is well received by authors, no matter their career stage. Yet, there seems to be the very real perception that this is not the case. Retaliations to referees in such a negative manner can represent serious cases of academic misconduct ( Fox, 1994; Rennie, 2003). It is important to note, however, that this is not a direct consequence of OPR, but instead a failure of the general academic system to mitigate and act against inappropriate behavior. Increased transparency can only aid in preventing and tackling the potential issues of abuse and publication misconduct, something which is almost entirely absent within a closed system. COPE provides advice to editors and publishers on publication ethics, and on how to handle cases of research and publication misconduct, including during peer review. The Committee on Publication Ethics (COPE) could continue to be used as the basis for developing formal mechanisms adapted to innovative models of peer review, including those outlined in this paper. Any new OPR ecosystem could also draw on the experience accumulated by Online Dispute Resolution (ODR) researchers and practitioners over the past 20 years. ODR can be defined as “the application of information and communications technology to the prevention, management, and resolution of disputes” ( Katsh & Rule, 2015), and could be implemented to prevent, mitigate, and deal with any potential misconduct during peer review alongside COPE. Therefore, the perceived danger of author backlash is highly unlikely to be acceptable in the current academic system, and if it does occur, it can be dealt with using increased transparency. Furthermore, bias and retaliation exist even in a double blind review process ( Baggs *et al.*, 2008; Snodgrass, 2007; Tomkins *et al.*, 2017), which is generally considered to be more conservative or protective. Such widespread identification of bias highlights this as a more general issue within peer review and academia, and we should be careful not to attribute it to any particular mode or trait of peer review. This is particularly relevant for more specialized fields, where the pool of potential authors and reviewers is relatively small ( Riggs, 1995). Nonetheless, careful evaluation of existing evidence and engagement with researchers, especially higher-risk or marginalized communities (e.g., Rodríguez-Bravo *et al.* (2017)), should be a necessary and vital step prior to implementation of any system of reviewer transparency. More training and guidance for reviewers, authors, and editors for their individual roles, expectations, and responsibilities also has a clear benefit here. One effort currently looking to address the training gap for peer review is the Publons Academy ( publons.com/community/academy/), although this is a relatively recent program and the effectiveness of it can not yet be assessed.

***2.4.3 The impact of identification and anonymity on bias.*** One of the biggest criticisms levied at peer review is that, like many human endeavours, it is intrinsically biased and not the objective and impartial process many regard it to be. Yet, the question is no longer about whether or not it is biased, but to what extent it is in different social dimensions - a debate which is very much ongoing (e.g., ( Lee *et al.*, 2013; Rodgers, 2017; Tennant, 2017)). One of the major issues is that peer review suffers from systemic confirmatory bias, with results that are deemed as significant, statistically or otherwise, being preferentially selected for publication ( Mahoney, 1977). This causes a distinct bias within the published research record ( van Assen *et al.*, 2014), as a consequence of perverting the research process itself by creating an incentive system that is almost entirely publication-oriented. Others have described the issues with such an asymmetric evaluation criteria as lacking the core values of a scientific process ( Bon *et al.*, 2017).

The evidence on whether there is bias in peer review against certain author demographics is mixed, but overwhelmingly in favor of systemic bias against women in article publishing ( Budden *et al.*, 2008; Darling, 2015; Grivell, 2006; Helmer *et al.*, 2017; Kuehn, 2017; Lerback & Hanson, 2017; Lloyd, 1990; McKiernan, 2003; Roberts & Verhoef, 2016; Smith, 2006; Tregenza, 2002) (although see also Blank (1991); Webb *et al.* (2008); Whittaker (2008)). After the journal *Behavioural Ecology* adopted double blind peer review in 2001, there was a significant increase in accepted manuscripts by women first authors; an effect not observed in similar journals that did not change their peer review policy ( Budden *et al.*, 2008). One of the most recent public examples of this bias is the case where a reviewer told the authors that they should add more male authors to their study ( Bernstein, 2015). More recently, it has been shown in the *Frontiers* journal series that women are under-represented in peer-review and that editors of both genders operate with substantial same-gender preference ( Helmer *et al.*, 2017). The most famous, but also widely criticised, piece of evidence on bias against authors comes from a study by Peters & Ceci (1982) using psychology journals. They took 12 published psychology studies from prestigious institutions and retyped the papers, making minor changes to the titles, abstracts, and introductions but changing the authors’ names and institutions. The papers were then resubmitted to the journals that had first published them. In only three cases did the journals realize that they had already published the paper, and eight of the remaining nine were rejected—not because of lack of originality but because of the perception of poor quality. Peters & Ceci (1982) concluded that this was evidence of bias against authors from less prestigious institutions, although the deeper causes of this bias remain unclear at the present. A similar effect was found in an orthopaedic journal by Okike *et al.* (2016), where reviewers were more likely to recommend acceptance when the authors’ names and institutions were visible than when they were redacted. Further studies have shown that peer review is substantially positively biased towards authors from top institutions ( Ross *et al.*, 2006; Tomkins *et al.*, 2017), due to the perception of prestige of those institutions and, consequently, of the authors as well. Further biases based on nationality and language have also been shown to exist ( Dall’Aglio, 2006; Ernst & Kienbacher, 1991; Link, 1998; Ross *et al.*, 2006; Tregenza, 2002).

While there are relatively few large-scale investigations of the extent and mode of bias within peer review (although see Lee *et al.* (2013) for an excellent overview), these studies together indicate that inherent biases are systemically embedded within the process, and must be accounted for prior to any further developments in peer review. This range of population-level investigations into attitudes and applications of anonymity, and the extent of any biases resulting from this, exposes a highly complex picture, and there is little consensus on its impact at a system-wide scale. However, based on these often polarised studies, it is inescapable to conclude that peer review is highly subjective, rarely impartial, and definitely not as homogeneous as it is often regarded.

Applying a single, blanket policy across the entire peer review system regarding anonymity would greatly degrade the ability of science to move forward, especially without a wide flexibility to manage exceptions. The reasons to avoid one definite policy are the inherent complexity of peer review systems, the interplay with different cultural aspects within the various sub-sectors of research, and the difficulty in identifying whether anonymous or identified works are objectively better. As a general overview of the current peer review ecosystem, Nobarany & Booth (2016) recently recommended that, due to this inherent diversity, peer review policies and support systems should remain flexible and customizable to suit the needs of different research communities. For example, some publishers allow authors to opt in to double blinded review Palus (2015), and others could expand this to offer a menu of peer review options. We expect that, by emphasizing the differences in shared values across research communities, we will see a new diversity of OPR processes developed across disciplines in the future. Remaining ignorant of this diversity of practices and inherent biases in peer review, as both social and physical processes, would be an unwise approach for future innovations.

### 2.5 Decoupling peer review from publishing

One proposal to transform scholarly publishing is to decouple the concept of the journal and its functions (e.g., archiving, registration and dissemination) from peer review and the certification that this provides. Some even regard this decoupling process as the “paradigm shift” that scholarly publishing needs ( Priem & Hemminger, 2012). Some publishers, journals, and platforms are now taking a more adventurous exploration of peer review that occurs subsequent to publication ( Figure 3). Here, the principle is that all research deserves the opportunity to be published (usually pending some form of initial editorial selectivity), and that filtering through peer review occurs subsequent to the actual communication of research articles (i.e., a publish then filter process). This is often termed “post-publication peer review,” a confusing terminology based on what constitutes “publication” in the digital age, depending on whether it occurs on manuscripts that have been previously peer reviewed or not ( blogs.openaire.eu/?p=1205), and a persistent academic view that published equals peer reviewed. Numerous venues now provide inbuilt systems for post-publication peer review, including *RIO*, *PubPub*, *ScienceOpen*, *The Winnower*, and *F1000 Research*. Some European Geophysical Union journals hosted on *Copernicus* offer a hybrid model with initial discussion papers receiving open peer review and comments and then selected papers accepted as final publications, which they term ‘Interactive Public Peer Review’ ( publications.copernicus.org/services/public_peer_review.html). Here, review reports are posted alongside published manuscripts, with an option for reviewers to reveal their identity should they wish ( Pöschl, 2012). In addition to the systems adopted by journals, other post-publication annotation and commenting services exist independent of any specific journal or publisher and operating across platforms, such as *hypothes.is*, *PaperHive*, and *PubPeer*.

Initiatives such as the *Peerage of Science*( peerageofscience.org), *RUBRIQ * ( rubriq.com), and *Axios Review* ( axiosreview.org; closed in 2017) have implemented decoupled models of peer review. These tools work based on the same core principles as traditional peer review, but authors submit their manuscripts to the platforms first instead of journals. The platforms provide the referees, either via subject-specific editors or via self-managed agreements. After the referees have provided their comments and the manuscript has been improved, the platform forwards the manuscript and the referee reports to a journal. Some journal policies accept the platform reviews as if the reviews were coming from the journal’s pool of reviewers, while others still require the journal’s handling editor to look for additional reviewers. While these systems usually cost money for authors, these costs can sometimes be deducted from any publication fees once the article has been published. Journals accept deduction of these costs because they benefit by receiving manuscripts that have already been assessed for journal fit and have been through a round of revisions, thereby reducing their workload. A consortium of publishers and commercial vendors recently established the Manuscript Exchange Common Approach (MECA; manuscriptexchange.org) as a form of portable review in order to cut down inefficiency and redundancy. Yet, it still is in too early a stage to comment on its viability.

*LIBRE* ( openscholar.org.uk/libre) is a free, multidisciplinary, digital article repository for formal publication and community-based evaluation. Reviewers’ assessments, citation indices, community ratings, and usage statistics, are used by *LIBRE* to calculate multiparametric performance metrics. At any time, authors can upload an improved version of their article or decide to send it to an academic journal. Launched in 2013, *LIBRE* was subsequently combined with the *Self-Journal of Science* ( sjscience.org) under the combined heading of *Open Scholar* ( openscholar.org.uk). One of the tools that *Open Scholar* offers is a peer review module for integration with institutional repositories, which is designed to bring research evaluation back into the hands of research communities themselves ( openscholar.org.uk/open-peer-review-module-for-repositories/). *Academic Karma* is another new service that facilitates peer review of preprints from a range of sources ( academickarma.org/).

***2.5.1 Preprints and overlay journals.*** In fields such as mathematics, astrophysics, or cosmology, research communities already commonly publish their work on the *arXiv* platform ( Larivière *et al.*, 2014). To date, *arXiv* has accumulated more than one million research documents – preprints or e-prints – and currently receives 8000 submissions a month with no costs to authors. *arXiv* also sparked innovation for a number of communication and validation tools within restricted communities, although these seem to be largely local, non-interoperable, and do not appear to have disrupted the traditional scholarly publishing process to any great extent ( Marra, 2017). In other fields, the uptake of preprints has been relatively slower, although it is gaining momentum with the development of platforms such as *bioRxiv* and several newly established ones through the *Center for Open Science*, including *engrXiv* ( engrXiv.org) and *psyarXiv* ( psyarxiv.com). Social movements such as ASAPBio ( asapbio.org) are helping to drive this expansion. Manuscripts submitted to these preprint servers are typically a draft version prior to formal submission to a journal for peer review, but can also be updated to include peer reviewed versions (often called post-prints). Primary motivation here is to bypass the lengthy time taken for peer review and formal publication, which means the timing of peer review occurs subsequent to manuscripts being made public. However, sometimes these articles are not submitted anywhere else and form what some regard as grey literature ( Luzi, 2000). Papers on digital preprint repositories are cited on a daily basis and much research builds upon them, although they may suffer from a stigma of not having the scientific stamp of approval of peer review ( Adam, 2010). Some journal policies explicitly attempt to limit their citation in peer-reviewed publications (e.g., *Nature* nature.com/nature/authors/gta/#a5.4), *Cell* cell.com/cell/authors), and recently the scholarly publishing sector even attempted to discredit their recognition as valuable publications ( asapbio.org/faseb). In spite of this, the popularity and success of preprints is testified by their citation records, with four of the top five venues in physics and maths being *arXiv* sub-sections ( scholar.google.com/citations?view_op=top_ venues&hl=en&vq=phy). Similarly, the single most highly cited venue in economics is the *NBER Working Papers * server ( scholar.google.com/citations?view_op=top_venues&hl=en&vq=bus_economics), according to the *Google Scholar* h5-index.

The overlay journal, first described by Ginsparg (1997) and built on the concept of deconstructed journals ( Smith, 1999), is a novel type of journal that operates by having peer review as an additional layer on top of collections of preprints ( Hettyey *et al.*, 2012; Patel, 2014; Stemmle & Collier, 2013; Vines, 2015b). New overlay journals such as *The Open Journal* ( theoj.org) or *Discrete Analysis* ( discreteanalysisjournal.com) are exclusively peer review platforms that circumvent traditional publishing by utilizing the pre-existing infrastructure and content of preprint servers like *arXiv*. Peer review is performed easily, rapidly, and cheaply, after initial publication of the articles. The reason they are termed “overlay” journals is that the articles remain on *arXiv* in their peer reviewed state, with the “journals” mostly comprising a simple list of links to these versions ( Gibney, 2016).

A similar approach to that of overlay journals is being developed by *PubPub* ( pubpub.org), which allows authors to self-publish their work. *PubPub* then provides a mechanism for creating overlay journals that can draw from and curate the content hosted on the platform itself. This model incorporates the preprint server and final article publishing into one contained system. *EPISCIENCES* is another platform that facilitates the creation of peer reviewed journals, with their content hosted on digital repositories ( Berthaud *et al.*, 2014). *ScienceOpen* provides editorially-managed collections of articles drawn from preprints and a combination of open access and non-open venues (e.g., scienceopen.com/collection/Science20). Editors compile articles to form a collection, write an editorial, and can invite referees to peer review the articles. This process is automatically mediated by *ORCID* for quality control (i.e., reviewers must have more than 5 publications associated with their ORCID profiles), and *CrossRef* and *Creative Commons* licensing for appropriate recognition. They are essentially equivalent to community-mediated overlay journals, but with the difference that they also draw on additional sources beyond preprints.

**Figure 4.**
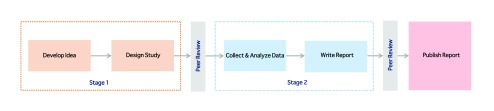
The publication process of Registered Reports. Each peer review stage also includes editorial input.

***2.5.2 Two-stage peer review and Registered Reports.*** Registered Reports represent a significant departure from conventional peer review in terms of relative timing and increased rigour ( Chambers *et al.*, 2014; Chambers *et al.*, 2017; Nosek & Lakens, 2014). Here, peer review is split into two stages. Research questions and methodology (i.e., the study design itself) are subject to a first round of evaluation prior to any data collection or analysis taking place ( Figure 4). Such a process is analogous to clinical trials registrations for medical research, the implementation of which became widespread many years before Registered Reports, and is a well-established specialised process that innovative peer review models could learn a lot from. If a protocol is found to be of sufficient quality to pass this stage, the study is then provisionally accepted for publication. Once the research has been finished and written-up, completed manuscripts are then subject to a second-stage of peer review which, in addition to affirming the soundness of the results, also confirms that data collection and analysis occurred in accordance with the originally described methodology. The format, originally introduced by the psychology journals *Cortex* and *Perspectives in Psychological Science* in 2013, is now used in some form by more than 70 journals ( Nature Human Behaviour, 2017) (see cos.io/rr/ for an up-to-date list of participating journals). Registered Reports are designed to boost research integrity by ensuring the publication of all research results, which helps reduce publication bias. As opposed to the traditional model of publication, where “positive” results are more likely to be published, results remain unknown at the time of the first review stage and therefore even “negative” results are equally as likely to be published. Such a process is designed to incentivize data-sharing, guard against dubious practices such as selective reporting of results (via so-called “p-hacking” and “HARKing”—Hypothesizing After the Results are Known) and low statistical power, and also prioritizes accurate reporting over that which is perceived to be of higher impact or publisher worthiness.

***2.5.3 Peer Review by Endorsement.*** A relatively new mode of named pre-publication review is that of pre-arranged and invited review, originally proposed as author-guided peer review ( Perakakis *et al.*, 2010), but now often called Peer Review by Endorsement (PRE). This has been implemented at *RIO*, and is functionally similar to the Contributed Submissions of *PNAS* ( pnas.org/site/authors/editorialpolicies.xhtml#contributed). This model requires an author to solicit reviews from their peers prior to submission in order to assess the suitability of a manuscript for publication. While some might see this as a potential bias, it is worth bearing in mind that many journals already ask authors who they want to review their papers, or who they should exclude. To avoid potential pre-submission bias, reviewer identities and their endorsements are made publicly available alongside manuscripts, which also removes any possible deleterious editorial criteria from inhibiting the publication of research. Also, PRE has been suggested by Jan Velterop to be much cheaper, legitimate, unbiased, faster, and more efficient alternative to the traditional publisher-mediated method ( theparachute.blogspot.de/2015/08/peer-review-by-endorsement.html. In theory, depending on the state of the manuscript, this means that submissions can be published much more rapidly, as less processing is required post-submission (e.g., in trying to find suitable reviewers). PRE also has the potential advantage of being more useful to non-native English speaking authors by allowing them to work with editors and reviewers in their first languages. However, possible drawbacks of this process include positive bias imposed by having author-recommended reviewers, as well as the potential for abuse through suggesting fake reviewers. As such, such a system highlights the crucial role of an Editor for verification and mediation.

***2.5.4 Limitations of decoupled peer review.*** Despite a general appeal for post-publication peer review and considerable innovation in this field, the appetite among researchers is limited, reflecting an overall lack of engagement with the process (e.g., Nature (2010)). Such a discordance between attitudes and practice is perhaps best exemplified in instances such as the “#arseniclife” debate. Here, a high profile but controversial paper was heavily critiqued in settings such as blogs and Twitter, constituting a form of social post-publication peer review, occurring much more rapidly than any formal responses in traditional academic venues ( Yeo *et al.*, 2017). Such social debates are notable, but however have yet to become mainstream beyond rare, high-profile cases.

As recently as 2012, it was reported that relatively few platforms allowed users to evaluate manuscripts post-publication ( Yarkoni, 2012). Even platforms such as *PLOS* have a restricted scope and limited user base: analysis of publicly available usage statistics indicate that at the time of writing, *PLOS* articles have each received an average of 0.06 ratings and 0.15 comments (see also Ware (2011)). Part of this may be due to how post-publication peer review is perceived culturally, with the name itself being anathema and considered an oxymoron, as most researchers usually consider a published article to be one that has already undergone formal peer review. At the present, it is clear that while there are numerous platforms providing decoupled peer review services, these are largely non-interoperable. The result of this, especially for post-publication services, is that most evaluations are difficult to discover, lost, or rarely available in an appropriate context or platform for re-use. To date, it seems that little effort has been focused on aggregating the content of these services (with exceptions such as *Publons*), which hinders its recognition as a valuable community process and for additional evaluation or assessment decisions.

While several new overlay journals are currently thriving, the history of their success is invariably limited, and most journals that experimented with the model returned to their traditional coupled roots ( Priem & Hemminger, 2012). Finally, it is probably worth mentioning that not a single overlay journal appears to have emerged outside of physics and math ( Priem & Hemminger, 2012). This is despite the fast growth of *arXiv* spin-offs like *biorXiv*, and potential layered peer review through services such as the recently launched *Peer Community In* ( peercommunityin.org).

*Axios Review* was closed down in early 2017 due to a lack of uptake from researchers, with the founder stating: “I blame the lack of uptake on a deep inertia in the researcher community in adopting new workflows” ( Davis, 2017). Combined with the generally low uptake of decoupled peer review processes, this suggests the overall reluctance of many research communities to adapt outside of the traditional coupled model. In this section, we have discussed a range of different arguments, variably successful platforms, and surveys and reports about peer review. Taken together, these reveal an incredible amount of friction to experimenting with peer review beyond that which is typically and incorrectly viewed as the only way of doing it. Much of this can be ascribed to tensions between evolving cultural practices, social norms, and the different stakeholder groups engaged with scholarly publishing. This reluctance is emphasized in recent surveys, for instance the one by Ross-Hellauer (2017) suggests that while attitudes towards the principles of OPR are rapidly becoming more positive, faith in its execution is not. We can perhaps expect this divergence due to the rapid pace of innovation, which has not led to rigorous or longitudinal evidence that these models are superior to the traditional process at either a population or system-wide level (although see Kovanis *et al.* (2017)). Cultural or social inertia, then, is defined by this cycle between low uptake and limited incentives and evidence. Perhaps more important is the general under-appreciation of this intimate relationship between social and technological barriers, that is undoubtedly required to overcome this cycle. The proliferation of social media over the last decade provides excellent examples of how digital communities can leverage new technologies for great effect.

## 3 Potential future models

As we have discussed in detail above, there has been considerable innovation in peer review in the last decade, which is leading to widespread critical examination of the process and scholarly publishing as a whole (e.g., ( Kriegeskorte *et al.*, 2012)). Much of this has been driven by the advent of Web 2.0 technologies and new social media platforms, and an overall shift towards a more open system of scholarly communication. Previous work in this arena has described features of a *Reddit*-like model, combined with additional personalized features of other social platforms, like *Stack Exchange*, *Netflix*, and *Amazon* ( Yarkoni, 2012). Here, we develop upon this by considering additional traits of models such as *Wikipedia*, *GitHub*, and *Blockchain*, and discuss these in the context of the rapidly evolving socio-technological environment for the present system of peer review. In the following section, we discuss potential future peer review platforms and processes in the context of the following three major traits, which any future innovation would greatly benefit from consideration of:

1. Quality control and moderation, possibly through openness and transparency;
2. Certification via personalized reputation or performance metrics;
3. Incentive structures to motivate and encourage engagement.

While discussing a number of principles that should guide the implementation of novel platforms for evaluating scientific work, Yarkoni (2012) argued that many of the problems researchers face have already been successfully addressed by a range of non-research focused social Web applications. Therefore, developing next-generation platforms for scientific evaluations should focus on adapting the best currently used approaches for these rather than on innovating entirely new ones ( Neylon & Wu, 2009; Priem & Hemminger, 2010; Yarkoni, 2012). One important element that will determine the success or failure of any such peer-to-peer reputation or evaluation system is a critical mass of researcher uptake. This has to be carefully balanced with the demands and uptakes of restricted scholarly communities, which have inherently different motivations and practices in peer review. A remaining issue is the aforementioned cultural inertia, which can lead to low adoption of anything innovative or disruptive to traditional workflows in research. This is a perfectly natural trait for communities, where ideas out-pace technological innovation, which in turn out-paces the development of social norms. Hence, rather than proposing an entirely new platform or model of peer review, our approach here is to consider the advantages and disadvantages of existing models and innovations in social services and technologies ( Table 4). We then explore ways in which such traits can be adapted, combined, and applied to build a more effective and efficient peer review system, while potentially reducing friction to its uptake.

**Table 4.** Potential pros and cons of the main features of the peer review models that are discussed. Note that some of these are already employed, alone or in combination, by different research platforms.

Feature	Description	Pros	Cons/Risks	Existing models
Voting or rating	Quantified review evaluation (5 stars, points), including up- and down-votes	Community-driven, quality filter, simple and efficient	Randomized procedure, auto-promotion, gaming, popularity bias, non-static	Reddit, Stack Exchange, Amazon
Openness	Public visibility of review content	Responsibility, accountability, context, higher quality	Peer pressure, potential lower quality, invites retaliation	All
Reputation	Reviewer evaluation and ranking (points, review statistics)	Quality filter, reward, motivation	Imbalance based on user status, encourages gaming, platform-specific	Stack Exchange, GitHub, Amazon
Public commenting	Visible comments on paper/ review	Living/organic paper, community involvement, progressive, inclusive	Prone to harassment, time consuming, non- interoperable, low re-use	Reddit, Stack Exchange, Hypothesis
Version control	Managed releases and configurations	Living/organic objects, verifiable, progressive, well- organized	Citation tracking, time consuming, low trust of content	GitHub, Wikipedia
Incentivization	Encouragement to engage with platform and process via badges/money or recognition	Motivation, return on investment	Research monetization, can be perverted by greed, expensive	Stack Exchange, Blockchain
Authentication and certification	Filtering of contributors via verification process	Fraud control, author protection, stability	Difficult to manage	Blockchain
Moderation	Filtering of inappropriate behavior in comments, rating	Community-driven, quality filter	Censorship, mainstream speech	Reddit, Stack Exchange

### 3.1 A Reddit-based model

*Reddit* ( reddit.com) is an open-source, community-based platform where users submit comments and original or linked content, organized into thematic lists of subreddits. As Yarkoni (2012) noted, a thematic list of subreddits can be automatically generated for any peer review platform using keyword metadata generated from sources like the National Library of Medicine’s Medical Subject Headings (MeSH). Members, or redditors, can upvote or downvote any submissions based on quality and relevance, and publicly comment on all shared content. Individuals can subscribe to contribution lists, and articles can be organized by time (newest to oldest) or level of engagement. Quality control is invoked by moderation through subreddit mods, who can filter and remove inappropriate comments and links. A score is given for each link and comment as the sum of upvotes minus downvotes, thus providing an overall ranking system. At *Reddit*, highly scoring submissions are relatively ephemeral, with an automatic down-voting algorithm implemented that shifts them further down lists as new content is added, typically within 24 hours of initial posting.

***3.1.1 Reddit as an existing “journal” of science.*** The subreddit for Science ( reddit.com/r/science) is a highly-moderated discussion channel, curated by at least 600 professional researchers and with more than 15 million subscribers at the time of writing. The forum has even been described as “The world’s largest 2-way dialogue between scientists and the public” ( Owens, 2014). Contributors here can add “flair” (a user-assigned tagging and filtering system) to their posts as a way of thematically organizing them based on research discipline, analogous to the container function of a typical journal. Individuals can also have flair as a form of subject-specific credibility (i.e., a peer status) upon provision of proof of education in their topic. Public contributions from peers are subsequently stamped with a status and area of expertise, such as “Grad student|Earth Sciences.”

Scientists already further engage with *Reddit* through science AMAs (Ask Me Anythings), which tend to be quite popular. However, the level of discourse provided in this is generally not equivalent in depth compared to that perceived for peer review, and is more akin to a form of science communication or public engagement with research. In this way, *Reddit* has the potential to drive enormous amounts of traffic to primary research and there even is a phenomenon known as the “ *Reddit* hug of death”, whereby servers become overloaded and crash due to *Reddit*-based traffic. The */r/science* subreddit is viewed as a venue for “scientists and lay audiences to openly discuss scientific ideas in a civilized and educational manner”, according to the organizer, Dr. Nathan Allen ( Lee, 2015). As such, an additional appeal of this model is that it could increase the public level of scientific literacy and understanding.

***3.1.2 Reddit-style peer evaluation.*** The essential part of any *Reddit*-style model with potential parallels to peer review is that links to scientific research can be shared, commented on, and ranked (upvoted or downvoted) by the community. All links or texts can be publicly discussed in terms of methods, context, and implications, similar to any scholarly post-publication commenting system. Such a process for peer review could essentially operate as an additional layer on top of a preprint archive or repository, much like a social version of an overlay journal. Ultimately, a public commenting system like this could achieve the same depth of peer evaluation as the formal process, but as a crowd-sourced process. However, it is important to note here that this is a mode of instantaneous publication prior to peer review, with filtering through interaction occurring post-publication. Furthermore, comments can receive similar treatment to submitted content, in that they can be upvoted, downvoted, and further commented upon in a cascading process. An advantage of this is that multiple comment threads can form on single posts and viewers can track individual discussions. Here, the highest-ranked comments could simply be presented at the top of the thread, while those of lowest ranking remain at the bottom.

In theory, a subreddit could be created for any sub-topic within research, and a simple nested hierarchical taxonomy could make this as precise or broad as warranted by individual communities. *Reddit* allows any user to create their own subreddit, pending certain status achievements through platform engagement. In addition, this could be moderated externally through *ORCID*, where a set number of published items in an ORCID profile are required for that individual to perform a peer review; or in this case, create a new subreddit. Connection to an academic profile within academia, such as *ORCID*, further allows community validation, verification, and judgement of importance. For example, being able to see whether senior figures in a given field have read or upvoted certain threads can be highly influential in decisions to engage with that thread, and vice versa. A very similar process already occurs at the *Self Journal of Science* ( sjscience.org/), where contributors have a choice of voting either “This article has reached scientific standards” or “This article still needs revisions”, with public disclosure of who has voted in either direction. Threaded commenting could also be implemented, as it is vital to the success of any collaborative filtering platform, and also provides a highly efficient corrective mechanism. Peer evaluation in this form emphasizes progress and research as a discourse over piecemeal publications or objects as part of a lengthier process. Such a system could be applied to other forms of scientific work, which includes code, data and images, thereby allowing contributors to claim credit for their full range of research outputs. Comments could be signed by default, pseudonymous, or anonymized until a contributor chooses to reveal their identity. If required, anonymized comments could be filtered out automatically by users. A key to this could be peer identity verification, which can be done at the back-end via email or integrated via *ORCID*.

***3.1.3 Translating engagement into prestige.*** *Reddit* karma points are awarded for sharing links and comments, and having these upvoted or downvoted by other registered members. The simplest implementation of such a voting system for peer review would be through interaction with any article in the database with a single click. This form of field-specific social recommendation for content simultaneously creates both a filter and a structured feed, similar to *Facebook* and *Google+*, and can easily be automated. With this, contributions get a rating, which accumulate to form a peer-based rating as a form of reputation and could be translated into a quantified level of community-granted prestige. Ratings are transparent and contributions and their ratings can be viewed on a public profile page. More sophisticated approaches could include graded ratings—e.g., five-point responses, like those used by *Amazon*—or separate rating dimensions providing peers with an immediate snapshot of the strengths and weaknesses of each article. Such a system is already in place at *ScienceOpen*, where referees evaluate an article for each of its importance, validity, completeness, and comprehensibility using a five-star system. For any given set of articles retrieved from the database, a ranking algorithm could be used to dynamically order articles on the basis of a combination of quality (an article’s aggregate rating within the system, like at *Stack Exchange*), relevance (using a recommendation system akin to *Amazon*), and recency (newly added articles could receive a boost). By default, the same algorithm would be implemented for all peers, as on *Reddit*. The issue here is making any such karma points equivalent to the amount of effort required to obtain them, and also ensuring that they are valued by the broader research community and assessment bodies. This could be facilitated through a simple badge incentive system, such as that designed by the *Center for Open Science* for core open practices ( cos.io/our-services/open-science-badges/).

***3.1.4 Can the wisdom of crowds work with peer review?*** One might consider a *Reddit*-style model as pitching quantity versus quality. Typically, comments provided on *Reddit* are not at the same level in terms of depth and rigor as those that we would expect from traditional peer review—as in, there is more to research evaluation than simply upvoting or downvoting. Furthermore, the range of expertise is highly variable due to the inclusion of specialists and non-specialists as equals (“peers”) within a single thread. However, there is no reason why a user prestige system akin to *Reddit* flair cannot be utilised to differentiate varying levels of expertise. The primary advantage here is that the number of participants is uncapped, therefore emphasizing the potential that *Reddit* has in scaling up participation in peer review. With a *Reddit* model, we must hold faith that sheer numbers will be sufficient in providing an optimal assessment of any given contribution and that any such assessment will ultimately provide a consensus of high quality and reusable results. Social review of this sort must therefore consider at what point is the process of review constrained in order to produce such a consensus, and one that is not self-selective as a factor of engagement rather than accuracy. This is termed the “Principle of Multiple Magnifications” by Kelty *et al.* (2008), which surmises that in spite of self-selectivity, more reviewers and more data about them will always be better than fewer reviewers and less data. The additional challenge here, then, will be to capture and archive consensus points for external re-use. Journals such as *F1000 Research* already have such a tagging system in place, where reviewers can mark a submission as approved after successive peer review iterations.

“The rich get richer” is one potential phenomenon for this style of system. Content from more prominent researchers may receive relatively more comments and ratings, and ultimately hype, as with any hierarchical system, including that for traditional scholarly publishing. Research from unknown authors may go relatively under-noticed and under-used, but will at least have been publicized. One solution to this is having a core community of editors, drawing on the r/science subreddit’s community of moderators. The editors could be empowered to invite peers to contribute to discussion threads, essentially wielding the same executive power as a journal editor, but combined with that of a forum moderator. Recent evidence suggests that such intelligent crowd reviewing has the potential to be an efficient and high quality process ( List, 2017).

### 3.2 An Amazon-style rate and review model

*Amazon* ( amazon.com/) was one of the first websites allowing the posting of public customer book reviews. The process is completely open to participation and informal, so that anyone can write a review and vote, providing usually that they have purchased the product. Customer reviews of this sort are peer-generated product evaluations hosted on a third-party website, such as *Amazon* ( Mudambi & Schuff, 2010). Here, usernames can be either real identities or pseudonyms. Reviews can also include images, and have a header summary. In addition, a fully searchable question and answer section on individual product pages allows users to ask specific questions, answered by the page creator, and voted on by the community. Top-voted answers are then displayed at the top. Chevalier & Mayzlin (2006) investigated the *Amazon* review system finding that, while reviews on the site tended to be generally more positive, negative reviews had a greater impact in determining sales. Reviews of this sort can therefore be thought of in terms of value addition or subtraction to a product or content, and ultimately can be used to help guide a third-party evaluation of a product and purchase decisions (i.e., a selectivity process).

***3.2.1 Amazon’s star-rating system.*** Star-rating systems are used frequently at a high-level in academia, and are commonly used to define research excellence, albeit perhaps in a flawed and an arguably detrimental way; e.g., the Research Excellence Framework in the UK ( ref.ac.uk) ( Mhurchú *et al.*, 2017; Moore *et al.*, 2017; Murphy & Sage, 2014). A study about Web 2.0 services and their use in alternative forms of scholarly communication by UK researchers found that nearly half (47%) of those surveyed expected that peer review would be complemented by citation and usage metrics and user ratings in the future ( Procter *et al.*, 2010a; Procter *et al.*, 2010b). *Amazon* provides an example of a sophisticated collaborative filtering system based on five-star user ratings, usually combined with several lines of comments and timestamps. Each product is summarized with the proportion of total customer reviews that have rated it at each star level. An average star rating is also given for each product. A low rating (one star) indicates an extremely negative view, whereas a high rating (five stars) reflects a positive view of the product. An intermediate scoring (three stars) can either represent a mid-view of a balance between negative and positive points, or merely reflect a nonchalant attitude towards a product. These ratings reveal fundamental details of accountability and are a sign of popularity and quality for items and sellers.

The utility of such a star-rating system for research is not immediately clear, or whether positive, moderate, or negative ratings would be more useful for readers or users. A superficial rating by itself would be a fairly useless design for researchers without being able to see the context and justification behind it. It is also unclear how a combined rate and review system would work for non-traditional research outputs, as the extremity and depth of reviews have been shown to vary depending on the type of content ( Mudambi & Schuff, 2010). Furthermore, the ubiquitous five-star rating tool used across the Web is flawed in practice and produces highly skewed results. For one, when people rank products or write reviews online, they are more likely to leave positive feedback. The vast majority of ratings on *YouTube*, for instance, is five stars and it turns out that this is repeated across the Web with an overall average estimated at about 4.3 stars, no matter the object being rated ( Crotty, 2009). Ware (2011) confirmed this average for articles rated in *PLOS*, suggesting that academic ranking systems operate in a similar manner to other social platforms. Rating systems also select for popularity rather than quality, which is the opposite of what scholarly evaluation seeks ( Ware, 2011). Another problem with commenting and rating systems is that they are open to gaming and manipulation. The *Amazon* system has been widely abused and it has been demonstrated how easy it is for an individual or small groups of friends to influence the popularity metrics even on hugely-visited websites like Time 100 ( Emilsson, 2015; Harmon & Metaxas, 2010). *Amazon* has historically prohibited compensation for reviews, prosecuting businesses who pay for fake reviews as well as the individuals who write them. Yet, with the exception that reviewers could post an honest review in exchange for a free or discounted product as long as they disclosed that fact. A recent study of over seven million reviews indicated that the average rating for products with these incentivized reviews was higher than non-incentivized ones ( Review Meta, 2016). Aiming to contain this phenomenon, *Amazon* has recently decided to adapt its Community Guidelines to eliminate incentivized reviews. As mentioned above, *ScienceOpen* offers a five-star rating system for articles, combined with post-publication peer review, but here the incentive is simply that the review content can be re-used, credited, and cited. Other platforms like *Publons* allow researchers to rate the quality of articles they have reviewed on a scale of 1–10 for both quality and significance. How such rating systems translate to user and community perception in an academic environment remains an interesting question for further research.

***3.2.2 Reviewing the reviewers.*** At *Amazon*, users can vote whether or not a review was helpful with simple binary yes or no options. Potential abuse can also be reported and avoided here by creating a system of community-governed moderation. After a sufficient number of yes votes, a user is upgraded to a spotlight reviewer through what essentially is a popularity contest. As a result, their reviews are given more prominence. Top reviews are those which receive the most helpful upvotes, usually because they provide more detailed information about a product. One potential way of improving rating and commenting systems is to weight such ratings according to the reputation of the rater (as done on *Amazon*, *eBay*, and *Wikipedia*). Reputation systems intend to achieve three things: foster good behavior, penalize bad behavior, and reduce the risk of harm to others as a result of bad behavior ( Ubois, 2003). Key features are that reputation can rise and fall and that reputation is based on behavior rather than social connections, thus prioritizing engagement over popularity. In addition, reputation systems do not have to use the true names of the participants but, to be effective and robust, they must be tied to an enduring identity infrastructure. Frishauf (2009) proposed a reputation system for peer review in which the review would be undertaken by people of known reputation, thereby setting a quality threshold that could be integrated into any social review platform and automated (e.g., via *ORCID*). One further problem with reputation systems is that having a single formula to derive reputation leaves the system open to gaming, as rationally expected with almost any process that can be measured and quantified. Gashler (2008) proposed a decentralized and secured system where each reviewer would digitally sign each paper, hence the digital signature would link the review with the paper. Such a web of reviewers and papers could be data mined to reveal information on the influence and connectedness of individual researchers within the research community. Depending on how the data were mined, this could be used as a reputation system or web-of-trust system that would be resistant to gaming because it would specify no particular metric.

### 3.3 A Stack Exchange/Overflow-style model

*Stack Exchange* ( stackexchange.com) is a collective intelligence system comprising multiple individual question and answer sites, many of which are already geared towards particular research communities, including maths and physics. The most popular site within *Stack Exchange* is *Stack Overflow*, a community of software developers and a place where professionals exchange problems, ideas, and solutions. *Stack Exchange* works by having users publish a specific problem or question, and then others contribute to a discussion on that issue. This format is considered to be a form of dynamic publishing by some ( Heller *et al.*, 2014). The appeal of *Stack Exchange* is that threaded discussions are often brief, concise, and geared towards solutions, all in a typical Web forum format. Highly regarded answers are positioned towards the top of threads, with others concatenated beneath. Like the *Amazon* model of weighted ratings, voting in *Stack Exchange* is more of a process that controls relative visibility. The result is a library of topical questions with high quality discussion threads and answers, developed by capturing the long tail of knowledge from communities of experts. The main distinction between this and scholarly publishing is that new material rarely is the focus of discussion threads. However, the ultimate goal remains the same: to improve knowledge and understanding of a particular issue. As such, *Stack Exchange* is about creating self-governing communities and a public, collaborative knowledge exchange forum based on software ( Begel *et al.*, 2013).

***3.3.1 Existing Overflow-style platforms.*** Some subject-specific platforms for research communities already exist that are similar to or based on *Stack Exchange* technology. These include *BioStars* ( biostars.org), a rapidly growing Bioinformatics resource, the use of which has contributed to the completion of traditional peer reviewed publications ( Parnell *et al.*, 2011). Another is *PhysicsOverflow*, an open platform for real-time discussions between the physics community combined with an open peer review system ( Pallavi Sudhir & Knöpfel, 2015) ( physicsoverflow.org/). *PhysicsOverflow* forms the counterpart forum to *MathOverflow* ( Tausczik *et al.*, 2014) ( https://mathoverflow.net/), with both containing a graduate-level question and answer forum, and an open problems section for collaboration on research issues. Both have a reviews section to complement formal journal-led peer review, where peers can submit preprints (e.g., from *arXiv*) for public peer evaluation, considered by most to be an “ *arXiv*-2.0”. Responses are divided into reviews and comments, and given a score based on votes for originality and accuracy. Similar to *Reddit*, there are moderators but these are democratically elected by the community itself. Motivation for engaging with these platforms comes from a personal desire to assist colleagues, progress research, and receive recognition for it ( Kubátová *et al.*, 2012) – the same as that for peer review for many. Together, both have created successful open community-led collaboration and discussion platforms for their research disciplines.

***3.3.2 Community-granted reputation and prestige.*** One of the key features of *Stack Exchange* is that it has an inbuilt community-based reputation system, karma, similar to that for *Reddit*. Identified peers rate or endorse the contributions of others and can indicate whether those contributions are positive (useful or informative) or negative. Karma provides a point-based reputation system for individuals, based not just on the quantity of engagement with the platform and its peers alone, but also on the quality and relevance of those engagements, as assessed by the wider engaging community ( stackoverflow.com/help/whats-reputation). Peers have their status and moderation privileges within the platform upgraded as they gain reputation. Such automated privilege administration provides a strong social incentive for constructively engaging within the community. Furthermore, peers who asked the original questions mark answers considered to be the most correct, thereby acknowledging the most significant contributions while providing a stamp of trustworthiness. This has the additional consequence of reducing the strain of evaluation and information overload for other peers by facilitating more rapid decision making, a behavior based on simple cognitive heuristics (e.g., social influences such as the “bandwagon effect” and position bias) ( Burghardt *et al.*, 2017). Threads can also be closed once questions have been answered sufficiently, based on a community decision, which enables maximum gain of potential karma points. This terminates further contribution but ensures that the knowledge is captured for future needs.

Karma and reputation can thus be achieved and incentivized by building and contributing to a growing community and providing knowledgeable and comprehensible answers on a specific topic. Within this system, reputation points are distributed based on social activities that are akin to peer review, such as answering questions, giving advice, providing feedback, sharing data, and generally improving the quality of work in the open. The points directly reflect an individual’s contribution to that specific research community. Such processes ultimately have a very low barrier to entry, but also expose peer review to potential gamification through integration with a reputation engine, a social bias which proliferates through any technoculture ( Belojevic *et al.*, 2014).

***3.3.3 Badge acquisition on Stack Overflow.*** An additional important feature of *Stack Overflow* is the acquisition of merit badges, which provide public stamps of group affiliation, experience, authority, identity and goal setting ( Halavais *et al.*, 2014). These badges define a way of locally and qualitatively differentiating between peers, and also symbolize motivational learning targets to achieve ( Rughiniş & Matei, 2013). *Stack Overflow* also has a system of tag badges to attribute subject-level expertise, awarded once a peer achieves a certain voting score. Together, these features open up a novel reputation system beyond traditional measurements based on publications and citations, that can also be used as an indication of expertise transferable beyond the platform itself. As such, a *Stack Exchange* model can increase the mobility of researchers who contribute in non-conventional ways (e.g., through software, code, teaching, data, art, materials) and are based at non-academic institutes. There is substantial scope in creating a reputation platform that goes beyond traditional measurements to include social network influence and open peer-to-peer engagement. Ultimately, this model can potentially transform the diversity of contributors to professional research and level the playing field for all types of formal contribution.

### 3.4 A GitHub-style model

*Git* is a free and open-source distributed version control system developed by the *Linux* community in 2005 ( git-scm.com/). *GitHub*, launched in 2008, works as a Web-based *Git* service and has become the de facto social coding platform for collaborative and open source development and code sharing ( Kosner, 2012; Thung *et al.*, 2013) ( github.com/). It holds many potentially desirable features that might be transferable to a system of peer review ( von Muhlen, 2011), such as its openness, version control and project management and collaborative functionalities, and system of accreditation and attribution for contributions. Despite its capability for not just sharing code, but also executable papers that automatically knit together text, data, and analyses into a living document, the true power of *GitHub* appears to be acknowledged infrequently by academic researchers ( Priem, 2013).

***3.4.1 Social functions of GitHub.*** Software review is an important part of software development, particularly for collaborative efforts. It is important that contributions are reviewed before they are merged into a code base, and *GitHub* provides this functionality. In addition, *GitHub* offers the ability to discuss specific issues, where multiple people can contribute to such a discussion, and discussions can refer to code segments or code changes and vice versa (but note that *GitHub* can also be used for non-code content). *GitHub* also includes a variety of notification options for both users and project repositories. Users can watch repositories or files of interest and be notified of any new issues or commits (updates), and someone who has discussed an issue can also be notified of any new discussions of that same issue. Issues can also be tagged (labelled in a manner that allows grouping of multiple issues with the same tag), and assigned to one or more participants, who are then responsible for that issue. Another item that *GitHub* supports is a checklist, a set of items that have a binary state, which can be used to implement and store the status of a set of actions. *GitHub* also allows users to form organizations as a way of grouping contributors together to manage access to different repositories. All contributions are made public as a way for users to obtain merit.

Prestige at *GitHub* can be further measured quantitatively as a social product through the star-rating system, which is derived from the number of followers or watchers and the number of times a repository has been forked (i.e., copied) or commented on. For scholarly research, this could ultimately shift the power dynamic in deciding what gets viewed and re-used away from editors, journals, or publishers to individual researchers. This then can potentially leverage a new mode of prestige, conferred through how work is engaged with and digested by the wider community and not by the packaging in which it is contained (analogous to the prestige often associated with journal brands).

Given these properties, it is clear that *GitHub* could be used to implement some style of peer evaluation and that it is well-suited to fine-grained iteration between reviewers, editors, and authors ( Ghosh *et al.*, 2012), given that all parties are identified. Making peer review a social process by distributing reviews to numerous peers, divides the burden and allows individuals to focus on their particular area of expertise. Peer review would operate more like a social network, with specific tasks (or repositories) being developed, distributed, and promoted through *GitHub*. As all code, data, and other content are supplied, and peers would be able to assess methods and results comprehensively, which in turn increases rigor, transparency, and replicability. Reviewers would also be able to claim credit and be acknowledged for their tracked contributions, and thereby quantify their impact on a project as a supply of individual prestige. This in turn facilitates the assessment of quality of reviews and reviewers. As such, evaluation becomes an interactive and dynamic process, with version control facilitating this all in a post-publication environment ( Ghosh *et al.*, 2012). The potential issue of proliferating non-significant work here is minimal, as projects that are not deemed to be interesting or of a sufficient standard of quality are simply never paid attention to in terms of follows, contributions, and re-use.

***3.4.2 Current use of GitHub for peer review.*** Two example uses of *GitHub* for peer review already exist in *The Journal of Open Source Software* ( *JOSS*; joss.theoj.org), created to give software developers a lightweight mechanism for software developers to quickly supplement their code with metadata and a descriptive paper, and then to submit this package for review and publication, and *ReScience* ( rescience.github.io), created to publish replication efforts in computational science.

The *JOSS* submission portal converts a submission into a new *GitHub* issue of type “pre-review” in the *JOSS*-review repository ( github.com/openjournals/joss-reviews). The editor-in-chief checks a submission, and if deemed suitable for review, assigns it to a topic editor who in turn assigns it to one or more reviewers. The topic editor then issues a command that creates a new issue of type “review”, with a check-list of required elements for the review. Each reviewer performs their review by checking off elements of the review issue with which they are satisfied. When they feel the submitter needs to make changes to make an element of the submission acceptable, they can either add a new comment in the review issue, which the submitter will see immediately, or they can create a new issue in the repository where the submitted software and paper exist—which could also be on *GitHub*, but is not required to be—and reference said issue in the review. In either case, the submitter is automatically and immediately notified of the issue, prompting them to address the particular concern raised. This process can iterate repeatedly, as the goal of *JOSS* is not to reject submissions but to work with submitters until their submissions are deemed acceptable. If there is a dispute, the topic editor (as well as the main editor, other topic editors, and anyone else who chooses to follow the issue) can weigh in. At the end of this process, when all items in the review check-list are resolved, the submission is accepted by the editor and the review issue is closed. However, it is still available and is linked from the accepted (and now published) submission. A good future option for this style of model could be to develop host-neutral standards using Git for peer review. For example, this could be applied by simply using a prescribed directory structure, such as: manuscript_version_1/peer_reviews, with open commenting via the issues function.

While *JOSS* uses *GItHub’*s issue mechanism, *ReScience* uses *GItHub’*s pull request mechanism: each submission is a pull request that is publicly reviewed and tested in order to guarantee that any researcher can re-use it. At least two reviewers evaluate and test the code and the accompanying material of a submission, continuously interacting with the authors through the pull request discussion section. If both reviewers can run the code and achieve the same results as were submitted by the author, the submission is accepted. If either reviewer fails to replicate the results before the deadline, the submission is rejected and authors are encouraged to resubmit an improved version later.

### 3.5 A Wikipedia-style model

*Wikipedia* is the freely available, multi-lingual, expandable encyclopedia of human knowledge ( wikipedia.org/). *Wikipedia*, like *Stack Exchange*, is another collaborative authoring and review system whereby contributing communities are essentially unlimited in scope. It has become a strongly influential tool in both shaping the way science is performed and in improving equitable access to scientific information, due to the ease and level of provision of information that it provides. Under a constant and instantaneous process of reworking and updating, new articles in hundreds of languages are added on a daily basis. *Wikipedia* operates through a system of collective intelligence based on linking knowledge workers through social media ( Kubátová *et al.*, 2012). Contributors to *Wikipedia* are largely anonymous volunteers, who are encouraged to participate mostly based on the principles guiding the platform (e.g., altruistic knowledge generation), and therefore often for reasons of personal satisfaction. Edits occur as cumulative and iterative improvements, and due to such a collaborative model, explicitly defining page-authorship becomes a complex task. Moderation and quality control is provided by a community of experienced editors and software-facilitated removal of mistakes, which can also help to resolve conflicts caused by concurrent editing by multiple authors ( wikipedia.org/wiki/Help:Edit_conflict). Platforms already exist that enable multiple authors to collaborate on a single document in real time, including *Google Docs*, *Overleaf*, and *Authorea*, which highlights the potential for this model to be extended into a wiki-style of peer review. *PLOS Computational Biology* is currently leading an experiment with Topic Pages ( collections.plos.org/topic-pages), which are published papers subsequently added as a new page to *Wikipedia* and then treated as a living document as they are enhanced by the community ( Wodak *et al.*, 2012). Communities of moderators on *Wikipedia* functionally exercise editorial power over content, and in principle anyone can participate, although experience with wiki-style operations is clearly beneficial. Other non-editorial roles, such as administrators and stewards, are nominated using conventional elections that variably account for their standing reputation. The apparent “free for all” appearance of *Wikipedia* is actually more of a sophisticated system of governance, based on implicitly shared values in the context of what is perceived to be useful for consumers, and transformed into operational rules to moderate the quality of content ( Kelty *et al.*, 2008).

***3.5.1 “Peers” and “reviews” in a wiki-world.*** *Wikipedia* already has its own mode of peer review, which anyone can request as a way to receive ideas on how to improve articles that are already considered to be “decent” ( wikipedia.org/wiki/Wikipedia:Peer_review/guidelines). It can be used for nominating potentially good articles that could become candidates for a featured article. Featured articles are considered to be the best articles *Wikipedia* has to offer, as determined by its editors and the fact that only ∼0.1% are selectively featured. Users submitting a new request are encouraged to review an article from those already listed, and encourage reviewers by replying promptly and appreciatively to comments. Compared to the conventional peer review process, where experts themselves participate in reviewing the work of another, the majority of the volunteers here, like most editors in *Wikipedia*, lack formal expertise in the subject at hand ( Xiao & Askin, 2012). This is considered to be a positive thing within the *Wikipedia* community, as it can help make technically-worded articles more accessible to non-specialist readers, demonstrating its power in a translational role for scholarly communication ( Thompson & Hanley, 2017).

When applied to scholarly topics, this process clearly lacks the “peer” aspect of scholarly peer review, which can potentially lead to propagation of factual errors (e.g., Hasty *et al.* (2014)). This creates a general perception of low quality from the research community, in spite of difficulties in actually measuring this ( Hu *et al.*, 2007). However, much of this perception can most likely be explained by a lack of familiarity with the model, and we might expect comfort to increase and attitudes to change with effective training and communications, and increased engagement and understanding of the process ( Xiao & Askin, 2014). If seeking expert input, users can invite editors from a subject-specific volunteers list or notify relevant WikiProjects. Furthermore, most *Wikipedia* articles never “pass” a review although some formal reviews do take place and can be indicated ( wikipedia.org/wiki/Category:Externally_peer_reviewed_articles). As such, although this is part of the process of conventional validation, such a system has little actual value on *Wikipedia* due to its dynamic nature. Indeed, wiki-communities appear to have distinct values to academic communities, being based more on inclusive community participation and mediation than on trust, exclusivity, and identification ( Wang & Wei, 2011). Verifiability remains a key element of the wiki-model, and has strong parallels with scholarly communication in fulfilling the dual roles of trust and expertise ( wikipedia.org/wiki/Wikipedia:Verifiability). Therefore, the process is perhaps best viewed as a process of “peer production”, but where attainment of the level of peer is relatively lower to that of an accredited expert. This provides a difference in community standing for *Wikipedia* content, with value being conveyed through contemporariness, mediation of debate, and transparency of information, rather than any perception of authority as with traditional scholarly works ( Black, 2008). Therefore, *Wikipedia* has a unique role in digital validation, being described as “not the bottom layer of authority, nor the top, but in fact the highest layer without formal vetting” ( chronicle.com/article/Wikipedia-Comes-of-Age/125899. Such a wiki-style process could be feasibly combined with trust metrics for verification, developed for sociology and psychology to describe the relative standing of groups or individuals in virtual communities ( ewikipedia.org/wiki/Trust_metric).

***3.5.2 Democratization of peer review.*** The advantage of *Wikipedia* over traditional review-then-publish processes comes from the fact that articles are enhanced consistently as new articles are integrated, statements are reworded, and factual errors are corrected as a form of iterative bootstrapping. Therefore, while one might consider a *Wikipedia* page to be of insufficient quality relative to a peer reviewed article at a given moment in time, this does not preclude it from meeting that quality threshold in the future. Therefore, *Wikipedia* might be viewed as an information trade-off between accuracy and scale, but with a gap that is consistently being closed as the overall quality generally improves. Another major statement that a *Wikipedia*-style of peer review makes is that rather than being exclusive, it is an inclusive process that anyone is allowed to participate in, and the barriers to entry are very low—anyone can potentially be granted peer status and participate in the debate and vetting of knowledge. This model of engagement also benefits from the “many eyes” hypothesis, where if something is visible to multiple people then, collectively, they are more likely to detect any errors in it, and tasks become more spread out as the size of a group increases. In *Wikipedia*, and to a larger extent *Wikidata*, automation or semi-automation through bots helps to maintain and update information on a large scale. For example, *Wikidata* is used as a centralized microbial genomics database ( Putman *et al.*, 2016), which uses bots to aggregate information from structured data sources. As such, *Wikipedia* represents a fairly extreme alternative to peer review where traditionally the barriers to entry are very high (based on expertise), to one where the pool of potential peers is relatively large ( Kelty *et al.*, 2008). This represents an enormous shift from the generally technocratic process of conventional peer review to one that is inherently more democratic. However, while the number of contributors is very large, more than 30 million, one third of all edits are made by only 10,000 people, just 0.03% ( wikipedia.org/wiki/Wikipedia:List_of_Wikipedians_by_number_of_edits). This is broadly similar to what is observed in current academic peer review systems, where the majority of the work is performed by a minority of the participants ( Fox *et al.*, 2017; Gropp *et al.*, 2017; Kovanis *et al.*, 2016).

One major implication of using a wiki-style model is the difference between traditional outputs as static, non-editable articles, and an output which is continuously evolving. As the wiki-model brings together information from different sources into one place, it has the potential to reduce redundancy compared to traditional research articles, in which duplicate information is often rehashed across many different locations. By focussing articles on new content just on those things that need to be written or changed to reflect new insights, this has the potential to decrease the systemic burden of peer review by reducing the amount and granularity of content in need of review. This burden is further alleviated by distributing the endeavor more efficiently among members of the wider community—a high-risk, high-gain approach to generating academic capital ( Black, 2008). Reviews can become more efficient, akin to those in software development, where they are focussed on units of individual edits, similar to the “commit” function in *GitHub* where suggested changes are recorded to content repositories. In circumstances where the granularity of the content to be added or changed does not fit with the wiki page in question, the material can be transferred to other pages, but the “original” page can still act as an information hub for the topic by linking to those other pages.

A possible risk with this approach is the creation of a highly conservative network of norms due to the governance structure, which could end up being even more bureaucratic and create community silos rather than coherence ( Heaberlin & DeDeo, 2016). To date, attempts at implementing a *Wikipedia*-like editing strategy for journals have been largely unsuccessful (e.g., at *Nature* ( Zamiska, 2006)). There are intrinsic differences in authority models used in *Wikipedia* communities (where the validity of the end result derives from verifiability, not personal authority of authors and reviewers) that would need to be aligned with the norms and expectations of research communities. In the latter, author statements and peer reviews are considered valid because of the personal, identifiable status and reputation of authors, reviews and editors, which could be feasibly combined with *Wikipedia* review models into a single solution. One example where this is beginning to happen already is with the WikiJournal User Group, which represents a publishing group of scholarly journals that apply academic peer review to their content ( meta.wikimedia.org/wiki/WikiJournal_User_Group). However, a more rigorous editorial review process is the reason why the original form of *Wikipedia*, known as *Nupedia*, ultimately failed ( Sanger, 2005). Future developments of any *Wikipedia*-like peer review tool could expect strong resistance from academic institutions due to potential disruption to assessment criteria, funding assignment, and intellectual property, as well as from commercial publishers, since academics would be releasing their research to the public for free instead of to them.

### 3.6 A Hypothesis-style annotation model

*Hypothesis* ( web.hypothes.is) is a lightweight, portable Web annotation tool that operates across publishing platforms ( Perkel, 2015), ambitiously described as a “peer review layer for the entire Internet” ( Farley, 2011). It relies on pre-existing published content to function, similar to other annotation services, such as *PubPeer* and *PaperHive*. Annotation is a process of enriching research objects through the addition of knowledge, and also provides an interactive educational opportunity by raising questions and creating opportunities to collect the perspectives of multiple peers in a single venue; providing a dual functionality for collaborative reading and writing. Web annotation services like *Hypothesis* allow annotations (such as comments or peer reviews) to live alongside the content but also separate from it, allowing communities to form and spread across the internet and across content types, such as HTML, PDF, EPUB, or other formats ( Whaley, 2017). Examples of such use in scholarly research already exist in post-publication peer review (e.g., Mietchen (2017)). Further, as of February 2017, annotation became a Web standard recognized by the Web Annotation Working Group, W3C (2017) (W3C). Under this model of Web annotation described by the W3C, annotations belong to and are controlled by the user rather than any individual publisher or content host. Users use a bookmarklet or browser extension to annotate any webpage they wish, and form a community of Web citizens.

*Hypothesis* permits the creation of public, group private, and individual private annotations, and is therefore compatible with a range of open and closed peer review models. Web annotation services not only extend peer review from academic and scholarly content to the whole Web, but open up the ability to annotate to any Web-browser. While the platform concentrates on focus groups within publishing, journalism, and academia, *Hypothesis* offers a new way to enrich, fact check, and collaborate on online content. Unlike *Wikipedia*, the original content never changes but the annotations are viewed as an overlay service on top of static content. This also means that annotations can be made at any time during the publishing process, including the preprint stage. Document Object Identifiers (DOIs) are used to federate or compile annotations for scholarly work. Reviewers often provide privately annotated versions of submitted manuscripts during conventional peer review, and Web annotation is part of the digitization of this process, while also decoupling it from journal hosts. A further benefit of Web annotations is that they are precise, since they can be applied in line rather than at the end of an article as is the case with formal commenting.

Annotations have the potential to enable new kinds of workflows where editors, authors, and reviewers all participate in conversations focussed on research manuscripts or other digital objects, either in a closed or public environment ( Vitolo *et al.*, 2015). At the present, activity performed by *Hypothesis* and other Web annotation services is poorly recognized in scholarly communities, although such activities can be tied to *ORCID*. However, there is definite value in services such as *PubPeer*, an online community mostly used for identifying cases of academic misconduct and fraud, perhaps best known for its user-led post-publication critique of a *Nature* paper on STAP (Stimulus-Triggered Acquisition of Pluripotency) cells. This ultimately prompted the formal retraction of the paper, demonstrating that post-publication annotation and peer review, as a form of self-correction and fraud detection, can out-perform that of the conventional pre-publication process. *PubPeer* has also been leveraged as a way to mass-report post-publication checks for the soundness of statistical analyses. One large-scale analysis using a tool called *statcheck* ( statcheck.io/ was used to post 50,000 annotations on the psychological literature ( Singh Chawla, 2016), as a form of large-scale public audit for published research.

### 3.7 A blockchain-based model

Peer review has the potential to be reinvented as a more efficient, fair, and otherwise attribute-enabled process through blockchains, a computer data structure that operates a distributed public ledger ( wikipedia.org/wiki/Blockchain). A blockchain connects a row of data blocks through a cryptographic function, with each block containing a time stamp and a link to the previous block in the chain. This system is decentralized, distributed, immutable, and transparent ( Antonopoulos, 2014; Nakamoto, 2008; Yli-Huumo *et al.*, 2016). Perhaps most importantly, individual chains are managed by peer-to-peer networks that collectively adhere to specific validation protocols. Blockchain became widely known as the data structure in Bitcoin due to its ability to efficiently record transactions between parties in a verifiable and permanent manner. It has also been applied to other uses including sharing verified business transactions, proof of ownership of legal documents, and distributed cloud storage.

The blockchain technology could be leveraged to create a tokenized peer review system involving penalties for members who do no uphold the adopted standards and vice versa. A blockchain-powered peer-reviewed journal could be issued as a token system to reward contributors, reviewers, editors, commentators, forum participants, advisors, staff, consultants, and indirect service providers involved in scientific publishing ( Swan, 2015). Such rewards could be in the form of reputation and/or remuneration, potentially through a form of digital currency (say *Science Coins*). Through a system of community trust, blockchains could be used to handle the following tasks:

1. Authenticating scientific papers (using time stamps and checksums), combating fraudulent science;
2. Allowing and encouraging reviewers to actively engage in the scientific community;
3. Rewarding reviewers for peer reviews with Science Coins;
4. Allowing authors to contribute by giving Science Coins;
5. Supporting verification and replicability of research.
6. Keeping reviewers and authors anonymous, while providing a validated certification of their identity as researchers, and rewarding them.

This could help to improve the quality and responsiveness of peer reviews, as these are published publicly and the different participants are rewarded for their contributions. For instance, reviewers for a blockchain-powered peer-reviewed journal could invest tokens in their comments and get rewarded if the comment is upvoted by other reviewers and the authors. All tokens need to be spent in making comments or upvoting other comments. When the peer review is completed, reviewers get rewarded according to the quality of their remarks. In addition, the rewards can be attributed even if reviewer and author identity is kept secret; such a system can decouple the quality assessment of the reviews from the reviews themselves, such that reviewers get credited while their reviews are kept anonymous. Moreover, increased transparency and interaction is facilitated between authors, reviewers, the scientific community, and the public. The journal *Ledger*, launched in 2015, is the first academic journal that makes use of a system of digital signatures and time stamps based on blockchain technology ( ledgerjournal.org). The aim is to generate irrevocable proof that a given manuscript existed on the date of publication. Another publishing platform being developed that leverages blockchain is *Aletheia*, which uses the technology to “achieve a distributed and tamper proof database of information, storing document metadata, vote topics, vote results and information specific to users such as reputation and certifications” ( github.com/aletheia-foundation/aletheia-whitepaper/blob/master/WHITE-PAPER.md#a-blockchain-journal).

Furthermore, blockchain-based models offer the potential to go well beyond peer review, possibly integrating all functions of publication in general. They could be used to support data publication, research evaluation, incentivization, and research fund distribution. A relevant example is a proposed decentralized peer review group as a way of managing quality control in peer review via blockchain through a system of cohort-based training ( Dhillon, 2016). This has also been leveraged as a “proof of existence” platform for scientific research ( Torpey, 2015) and medical trials ( Carlisle, 2014). However, the uptake from the academic community remains low thus far, despite claims that it could be a potential technical fix to the reproducibility crisis in research ( Bartling & Fecher, 2016). As with other novel processes, this is likely due to broad-scale unfamiliarity with blockchain, and perhaps even discomfort due to its financial association with Bitcoin.

### 3.8 AI-assisted peer review

Another frontier is the advent and growth of natural language processing, machine learning (ML), and neural network tools that may potentially assist with the peer review process. ML, as a technique, is rapidly becoming a service that can be utilized at a low cost by an increasing number of individuals. For example, *Amazon* now provides ML as a service through their *Amazon Web Services* platform ( aws.amazon.com/amazon-ai/), *Google* released their open source ML framework, *TensorFlow* ( tensorflow.org/), and *Facebook* have similarly contributed code of their *Torch* scientific learning framework ( torch.ch/). ML has been very widely adopted in tackling various challenges, including image recognition, content recommendation, fraud detection, and energy optimization. In higher education, adoption has been limited to automated evaluation of teaching and assessment, and in particular for plagiarism detection. The primary benefits of Web-based peer assessment are limiting peer pressure, reducing management workload, increasing student collaboration and engagement, and improving the understanding of peers as to what critical assessment procedures involve ( Li *et al.*, 2009).

The same is approximately true for using computer-based automation for peer review, for which there are three main practical applications. The first is determining whether a piece of work under consideration meets the minimal requirements of the process to which it has been submitted (i.e., for recommendation). For example, does a clinical trial contain the appropriate registration information, are the appropriate consent statements in place, have new taxonomic names been registered, and does the research fit in with the existing body of published literature ( Sobkowicz, 2008). The computer might also look at consistency through the paper; for example searching for statistical error or method description incompleteness: if there is a multiple group comparison, whether the p-value correction algorithm is indicated. This might be performed using a simpler text mining approach, as is performed by *statcheck* ( Singh Chawla, 2016). Under normal technical review these criteria need to be (or should be) checked manually either at the editorial submission stage or at the review stage. ML techniques can automatically scan documents to determine if the required elements are in place, and can generate an automated report to assist review and editorial panels, facilitating the work of the human reviewers. Moreover, any relevant papers can be automatically added to the editorial request to review, enabling referees to automatically have a greater awareness of the wider context of the research. This could also aid in preprint publication before manual peer review occurs.

The second approach is to automatically determine the most appropriate reviewers for a submitted manuscript, by using a co-authorship network data structure ( Rodriguez & Bollen, 2008). The advantage of this is that it opens up the potential pool of referees beyond who is simply known by an editor or editorial board, or recommended by authors. Removing human-intervention from this part of the process reduces potential biases (e.g., author recommended exclusion or preference) and can automatically identify potential conflicts of interest ( Khan, 2012). Dall’Aglio (2006) suggested ways this algorithm could be improved, for example through cognitive filtering to automatically analyze text and compare that to editor profiles as the basis for assignment. This could be built upon for referee selection by using an algorithm based on social networks, which can also be weighted according to the influence and quality of participant evaluations ( Rodriguez *et al.*, 2006), and referees can be further weighted based on their previous experience and contributions to peer review and their relevant expertise, thereby providing a way to train and develop the identification algorithm.

Thirdly, given that machine-driven research has been used to generate substantial and significant novel results based on ML and neural networks, we should not be surprised if, in the future, they can have some form of predictive utility in the identification of novel results during peer review. In such a case, machine learning would be used to predict the future impact of a given work (e.g., future citation counts), and in effect to do the job of impact analysis and decision making instead of or alongside a human reviewer. We have to keep a close watch on this potential shift in practice as it comes with obvious potential pitfalls by encouraging even more editorial selectivity, especially when network analysis is involved. For example, research in which a low citation future is predicted would be more susceptible to rejection, irrespective of the inherent value of that research. Conversely, submissions with a high predicted citation impact would be given preferential treatment by editors and reviewers. Caution in any pre-publication judgements of research should therefore always be adopted, and not be used as a surrogate for assessing the real world impact of research through time. Machine learning is not about providing a total replacement for human input to peer review, but more how different tasks could be delegated or refined through automation.

Some platforms already incorporate such AI-assisted methods for a variety of purposes. *Scholastica* ( scholasticahq.com) includes real-time journal performance analytics that can be used to assess and improve the peer review process. *Elsevier* uses a system called Evise ( elsevier.com/editors/evise) to check for plagiarism, recommend reviewers, and verify author profile information by linking to *Scopus*. The *Journal of High Energy Physics* uses automatic assignment to editors based on a keyword-driven algorithm ( Dall’Aglio, 2006). This process has the potential to be entirely independent from journals and can be easily implemented as an overlay function for repositories, including preprint servers. As such, it can be leveraged for a decoupled peer review process by combining certification with distribution and communication. It is entirely feasible for this to be implemented on a system-wide scale, with researcher databases such as *ORCID* becoming increasingly widely adopted. However, as the scale of such an initiative increases, the risk of over-fitting also increases due to the inherent complexity in modelling the diversity of research communities, although there are established techniques to avoid this. Questions have been raised about the impact of such systems on the practice of scholarly writing, such as how authors may change their approach when they know their manuscript is being evaluated by a machine ( Hukkinen, 2017), or how machine assessment could discover unfounded authority in statements by authors through analysis of citation networks ( Greenberg, 2009). One additional potential drawback of automation of this sort is the possibility for detection of false positives that might discourage authors from submitting.

Finally, it is important to note that ML and neural networks are largely considered to be conformist, so they have to be used with care ( Szegedy *et al.*, 2014), and perhaps only for recommendations rather than decision making. The question is not about whether automation produces error, but whether it produces less error than a system solely governed by human interaction. And if it does, how does this factor in relation to the benefits of efficiency and potential overhead cost reduction? Nevertheless, automation can potentially resolve many of the technical issues associated with peer review and there is great scope for increasing the breadth of automation in the future. Initiatives such as *Meta*, an AI tool that searches scientific papers to predict the trajectory of research ( meta.com), highlight the great promise of artificial intelligence in research and for application to peer review.

### 3.9 Peer review for non-text products

The focus of this article has focused on peer review for traditional text-based scholarly publications. However, peer review has also evolved to a wider variety of research outputs, policies, processes, and even people. These non-text products are increasingly being recognized as important intellectual contributions to the research ecosystem. While it is beyond the scope of the present paper to discuss all different modes of peer review, we discuss it briefly here in the context of software in order to note the similarities and differences, and to stimulate further investigation of the diversity of peer review processes (e.g., Penev *et al.* (2017)).

In order for the creators (authors) of non-traditional products to receive academic credit, they must currently be integrated into the publication system that forms the basis for academic assessment and evaluation. Peer review of methodologies, such as *protocols.io* ( protocols.io), allows for detailed OPR of methods while also promoting reproducibility and refinement of techniques. This can help other scholars to begin work on related projects and test methodologies due to the openness of both the protocols themselves and the comments on them ( Teytelman *et al.*, 2016). Digital humanities projects, which include visualizations, text processing, mapping, and many other varied outputs, have been a subject for re-evaluating the role of peer review, especially for the purpose of tenure and evaluation ( Ball *et al.*, 2016). In 2006, the *Modern Languages Association* released a statement on the peer review and evaluation of new forms of scholarship, insisting that they “be assessed with the same rigor used to judge scholarly quality in print media” ( Stanton *et al.*, 2007). Fitzpatrick (2011a) considered the idea of an objective evaluation of non-text products in the humanities, as well as the challenges faced during evaluation of a digital product that may have much more to review than a traditional text product, including community engagement and sustainability practices. To work with these non-text products, humanities scholars have used multiple methods of peer review and embraced OPR in order to adapt to the increased creation of non-text, multimedia scholarly products, and to integrate these products into the scholarly record and review process ( Anderson & McPherson, 2011).

***3.9.1 Software peer review.*** Software represents another area where traditional peer review has evolved. In software, peer review of code has been a standard part in computationally-intensive research for many years, particularly as a post-software creation check. Additionally, peer-programming (also known as pair-programming) has been growing in popularity, especially as part of the Agile methodology, where it is employed as a check made during software creation ( Lui & Chan, 2006). Software development and sharing platforms, such as *GitHub*, support and encourage social code review, which can be viewed as a form of peer review that takes place both during creation and afterwards. However, developed software has not traditionally been considered an academic product for the purpose of hiring, tenure, and promotion. Likewise, this form of evaluation has not been formally recognized as peer review by the academic community yet.

When it comes to software development, there is a dichotomy of review practices. On one hand, software developed in open source communities (not all software is released as open source; some is kept as proprietary for commercial reasons) relies on peer review as an intrinsic part of its existence, from creation and through continual evolution. On the other hand, software created in academia is typically not subjected to the same level of scrutiny. For the most part, at present there is no requirement for software used to analyze and present data in scholarly publications to be released as part of the publication process, let alone be closely checked as part of the review process, though this may be changing due to government mandates and community concerns about reproducibility. One example from Computer Science is ACM SIGPLAN’s Programming Language Design and Implementation conference that encourages the submission of supporting material (including code) for review by a separate technical committee. Papers with successfully evaluated artifacts get stamped with seals of approval visible in the conference proceedings. ACM is adopting a similar strategy on a wider scale through its Task Force on Data, Software, and Reproducibility in Publication ( acm.org/data-software-reproducibility). Academic code is sometimes released as open source, and many such released codebases have led to remarkable positive changes, with prominent examples including the Berkeley Software Distribution (BSD), upon which the Mac operating system (MacOS) is built; the ubiquitous TCP/IP Internet protocol stack; the Squid web proxy; the Xen hypervisor, which underpins many cloud computing infrastructures; Spark, the big data stream processing framework; and the Weka machine learning suite.

In order to gain recognition for their software work, authors initially made as few changes to the existing system as possible and simply wrote traditional papers about their software, which became acceptable in an increasing number of journals over time (see the extensive list compiled by the UK’s Software Sustainability Institute: software.ac.uk/which-journals-should-i-publish-my-software). At first, peer review for these software articles was the same as for any other paper, but this is changing now, particularly as journals specializing in software (e.g., *SoftwareX *( journals.elsevier.com/softwarex), the *Journal of Open Research Software *( *JORS*, openresearchsoftware.metajnl.com), the *Journal of Open Source Software* ( *JOSS*, joss.theoj.org)) are emerging. The material that is reviewed for these journals is both the text and the software. For *SoftwareX *( elsevier.com/authors/author-services/research-elements/software-articles/original-software-publications#submission) and *JORS* ( openresearchsoftware. metajnl.com/about/#q4), the text and the software are reviewed equally. For *JOSS*, the review process is more focused on the software (based on the *rOpenSci* model ( Ross *et al.*, 2016) and less on the text, which is intended to be minimal ( joss.theoj.org/about#reviewer_guidelines). The purpose of the review also varies across these journals. In *SoftwareX* and *JORS*, the goal of the review is to decide if the paper is acceptable and to improve it through a non-public editor-mediated iteration with the authors and the anonymous reviewers. While in *JOSS*, the goal is to accept most papers after improving them if needed, with the reviewers and authors ideally communicating directly and publicly through *GitHub* issues. Although submitting source code is still not required for most peer review processes, attitudes are slowly changing. As such, authors increasingly publish works presented at major conferences (which are the main channel of dissemination in computer science) as open source, and also increasingly adopting the use of *arXiv* as a publication venue ( Sutton & Gong, 2017).

### 3.10 Using multiple peer review models

While individual publishers may use specific methods when peer review is controlled by the author of the document to be reviewed, multiple peer review models can be used either in series or in parallel. For example, the *FORCE11* Software Citation Working Group used three different peer review models and methods to iteratively improve their principles document, leading to a journal publication ( Smith *et al.*, 2016). Initially, the document that was produced was made public and reviewed by *GitHub* issues ( github.com/force11/force11-scwg [see Section 3.4]). The next version of the document was placed on a website, and new reviewers commented on it both through additional *GitHub* issues and through *Hypothesis *( via.hypothes.is/https://www.force11.org/software-citation-principles [see Section 3.6]). Finally, the document was submitted to *PeerJ Computer Science*, which used a pre-publication review process that allowed reviewers to sign their reviews and the reviews to be made public along with the paper authors’ responses after the final paper was accepted and published ( Klyne, 2016; Kuhn, 2016a; Kuhn, 2016b). The authors also included an appendix that summarized the reviews and responses from the second phase. In summary, this document underwent three sequential and non-conflicting review processes and methods, where the second one was actually a parallel combination of two mechanisms. Some text-non-text hybrids platforms already exist that could leverage multiple review types; for example, *Jupyter notebooks* between text, software and data ( jupyter.org/), or traditional data management plans for review between text and data. Using such hybrid evaluation methods could prove to be quite successful, not just for reforming the peer review process, but also to improve the quality and impact of scientific publications. One could envision such a hybrid system with elements from the different models we have discussed.

## 4 A hybrid peer review platform

In Section 3, we summarized a range of social and technological traits of a range of individual existing social platforms. Each of these can, in theory, be applied to address specific social or technical criticisms of conventional peer review, as outlined in Section 2. Many of them are overlapping and can be modeled into, and leveraged for, a single hybrid platform. The advantage is that they each relate to the core non-independent features required for any modern peer review process or platform: *quality control*, *certification*, and *incentivization*. Only by harmonizing all three of these, while grounding development in diverse community stakeholder engagement, can the implementation of any future model of peer review be ultimately successful. Such a system has the potential to greatly disrupt the current coupling between peer review and journals, and lead to an overhaul of digital scholarly communication to become one that is fit for the modern research environment.

### 4.1 Quality control and moderation

Quality control is often hailed as the core function of peer review, but is invariably difficult to measure. Typically, it has been administered in a closed system, where editorial management formed the basis. A strong coupling of peer review to journals plays an important part in this, due to the association of researcher prestige with journal brand as a proxy for quality. By looking at platforms such as *Wikipedia* and *Reddit*, it is clear that community self-organization and governance represent a possible alternative when combined with a core community of moderators. These moderators would have the same operational functionality as editors in terms of gate-keeping and facilitating the process of engagement, but combined with the role of a Web forum moderator. Research communities could elect groups of moderators based on expertise, prior engagement with peer review, and transparent assessment of their reputation. This layer of moderation could be fully transparent in terms of identity by using persistent identifiers such as *ORCID*. The role of such moderators could be essentially identical to that of journal editors, in soliciting reviews from experts, making sure there is an even spread of review attention, and mediating discussions. Different communities could have different norms and procedures to govern content and engagement, and to self-organize into individual but connected platforms, similar to *Stack Exchange* or *Reddit*. *ORCID* has a further potential role of providing the possibility for a public archive of researcher information and metadata (e.g., publishing histories) that can be leveraged using automated techniques to match potential referees to items of interest, while avoiding conflicts of interest.

In such a system, published objects could be preprints, data, code, or any other digital research output. If these are combined with management through version control, similar to *GitHub*, quality control is provided through a system of automated but managed invited review, public interaction and collaboration (like with *Stack Exchange*), and transparent refinement. This would also help prevent a situation where “the rich get richer”, as semi-automation ensures that all content has the same chance of being interacted with. Engagement could be conducted via a system of issues and public comments, as on *GitHub*, where the process is not to reject submissions, but to provide a system of constant improvement. Such a system is already implemented successfully at *JOSS*. Both community moderation and crowd sourcing would play an important role here to prevent underdeveloped feedback that is not constructive and could delay efficient manuscript progress. This could be further integrated with a blockchain process so that each addition to the process is transparent and verifiable.

When authors and moderators deem the review process to have been sufficient for an object to have reached a community-decided level of quality or acceptance, threads can be closed (but remain public with the possibility of being re-opened, similar to *GitHub* issues), indexed, and the latest version is assigned a persistent identifier, such as a *CrossRef* DOI, as well as an appropriate license. If desired, these objects could then form the basis for submissions to journals, perhaps even fast-tracking them as the communication and quality control would already have been completed. Such a process would promote inclusive participation, community interaction, and quality would become a transparent function of how information is engaged with, digested, and reused. The role of peer review would then be coupled with the concept of a “living published unit”, independent of journals themselves. The role of journals and publishers would be dependent on how well they justify their added value, once community-wide and public dissemination and peer review have been decoupled from them.

### 4.2 Certification and reputation

The current peer review process is generally poorly recognized as a scholarly activity. It remains quite imbalanced between publishers who receive financial gain for organising it and researchers who receive little or no compensation for performing it. Opacity in the peer review process provides a way for others to capitalize on it, as this provides a mechanism for those managing it, rather than performing it, to take credit in one form or another. This explains at least in part why there is resistance from many publishers in providing any form of substantive recognition to peer reviewers. Exposing the process, decoupling it from journals and providing appropriate recognition to those involved helps to return peer review to its synergistic, intra-community origin. Performance metrics provide a way of certifying the peer review process, and provide the basis for incentivizing engagement. As outlined above, a fully transparent and interactive process of engagement combined with reviewer identification exposes the level of engagement and the added value from each participant.

Certification can be provided to referees based on the nature of their engagement with the process: community evaluation of their contributions (e.g. *Amazon*, *Reddit*, or *Stack Exchange*), combined with their reputation as authors. Rather than having anonymous or pseudonymous participants, for peer review to work well, it would require full identification, to connect on-platform reputation and authorship history. Rather than a journal-based form, certification is granted based on continuing engagement with the research process and is revealed at the article (or object) and individual level. Communities would need to decide whether or not to set engagement filters based on quantitative measures of experience or reputation, and what this should be for different activities. This should be highly appealing not just to researchers, but also to those in charge of hiring, tenure, promotion, grant funding, ethical review and research assessment, and therefore could become an important factor in future policy development. Models like *Stack Exchange* are ideal candidates for such a system, because achievement of certification takes place via a process of community engagement and can be quantified through a simple and transparent up-voting and down-voting scheme, combined with achievement badges. Any outputs from assessment could be portable and applied to *ORCID* profiles, external webpages, and continuously updated and refined through further activity. While a star system does not seem appealing due to the inherent biases associated with it, this quantitative way of “reviewing the reviewers” creates a form of dynamic social reputation. As this is decoupled from journals, it alleviates all of the well-known issues with journal-based ranking systems (e.g., Brembs *et al.* (2013)) and is fully transparent. By combining this with moderation, as outlined above, gaming can also be prevented (e.g., by providing numerous low quality engagements). Integrating a blockchain-based token system could also reduce potential for such gaming. Most importantly though, is that the research communities, and engagement within them, form the basis of certification, and reputation should evolve continuously with this.

### 4.3 Incentives for engagement

Incentives are broadly seen to be required to motivate and encourage wider participation and engagement with peer review. As such, this requires finding the sweet spot between lowering the threshold of entry for different research communities, while providing maximum reward. One of the most widely-held reasons for researchers to perform peer review is a shared sense of academic altruism or duty to their respective community (e.g., Ware (2008)). Despite this natural incentive to engage with the process, it is still clear that the process is imbalanced and researchers feel that they still receive far too little credit as a way of recognizing their efforts. Incentives, therefore, need not just encourage engagement with peer review, but with it in a way that is of most value to research communities through high quality, constructive feedback. This then demands transparency of the process, and becomes directly tied to certification and reputation, as above, which is the ultimate goal of any incentive system.

New ways of incentivizing peer review can be developed by quantifying engagement with the process and tying this in to academic profiles, such as *ORCID*. To some extent this is already performed via *Publons*, where the records of individuals reviewing for a particular journal can be integrated into *ORCID*. This could easily be extended to include aspects from *Reddit*, *Amazon*, and *Stack Exchange*, where participants receive virtual rewards, such as points or karma, for engaging with peer review and having those activities further evaluated and ranked by the community. After a certain quantified threshold has been achieved, a hierarchical award system could be developed into this, and then be subsequently integrated into *ORCID*. Such awards or badges could include “Top reviewer”, “Verified reviewer”, “Community leader’,’ or whatever individual communities decide is best for them. This can form an incentive loop, where additional engagement abilities are acquired based on achievement of such badges.

Highly-rated reviews gain more exposure and more credit, thus there incentive is to engage with the process in a way that is most beneficial to the community. Engagement with peer review and community evaluation of that then becomes part of a verified academic record, which can then be used as a way of establishing individual prestige. Such a system would be automatically integrated with any published content itself and objects could be similarly granted badges, such as “Community reviewed,” “Community accepted,” or “500 upvotes” as a way of quantifying the process. Therefore, there would be a dual incentive for authors to maximize engagement from the research community and for that community to productively engage with content. A potential extension of this in the form of monetization (e.g., through a blockchain protocol) is perhaps unwise, as it might lead to a distortion of incentives.

### 4.4 Challenges and future work

None of the ideas we have proposed here are particularly radical, representing more the recombination of existing variants that have succeeded or failed to varying degrees. We have presented them here in the context of historical developments and current criticisms of peer review in the hope that they inspire further discussion and innovation. A key challenge that our proposed hypothetical hybrid system will have to overcome is simultaneous uptake across the whole scholarly ecosystem. This in turn will most likely require substantial evidence that such an alternative system is more effective than the traditional processes (e.g., Kovanis *et al.* (2017)), which, as discussed in this article, is problematic in design and execution. Furthermore, this proposed system involves a requirement for standardised communication between a range of key participants. Real shifts will occur where elements of this system can be taken up by specific communities, and remain interoperable between them. At the present, it remains unclear as to how these communities should be formed, and what the role of existing structures including learned societies, and institutes and labs from across different geographies, could be. Strategically identifying sites where stepwise changes in practice are desirable to a community is an important next step, but will be important in addressing the challenges in reviewer engagement and recognition. Increasing the almost non-existent current role and recognition of peer review in promotion, hiring and tenure processes could be a critical step forward for incentivizing the changes we have discussed. However, it is also clear that recent advances in technology can play a significant role in systemic changes to peer review. High quality implementations of these ideas in systems that communities can choose to adopt may act as *de facto* standards that help to build towards consistent practice and adoption.

The Internet has changed our expectations of how communication works, and enabled a wide array of new, technologically-enabled possibilities to change how we communicate and interact online. Peer review has also recently become an online endeavor, but few organizations who conduct peer review have adopted Internet-style communication norms. This leaves a gap in what is possible with current technology and social norms and what we are doing to ensure the reliability and trustworthiness of published science. Peer review is a critical part of an effective scientific enterprise, but many of those who conduct peer review and depend upon it do not fully understand the theoretical and empirical basis for it. This means that our efforts to advance and change peer review are being driven by organizational goals such as market position and profit, and not by the needs of academia.

Existing, popular online communication systems and platforms were designed to attract a huge following, not to ensure the ethics and reliability of effective peer review. Numerous front-end Web applications already implement all of the essential core traits for creating a widely distributed, diverse peer review ecosystem. We already have the technology we need. However, it will take a lot of work to integrate new technology-mediated communication norms into effective, widely-accepted peer review models, and connect these together seamlessly so that they become inter-operable as part of a sustainable scholarly communications infrastructure. Identity is a core factor driving online communication adoption and social norms and practices of current peer review – both how it is traditionally conducted with editorial management, and what will be possible with novel models online.

These socio-technological barriers cannot be overcome by simply creating platforms and expecting researchers to use them – the “if you build it, they will come” fallacy. Rather, as others have suggested (e.g., Moore *et al.* (2017); Prechelt *et al.* (2017)), platforms should be developed with community engagement, education, and capacity building as core traits, in order to help understand the cultural processes and needs of different disciplines and create solutions around those. Coordinated efforts are required to teach and market the purpose of peer review to researchers. More effective engagement is clearly required to emphasize the distinction between the idealized processes of peer review, along with the perceptions and applications of it, and the resulting products and services available to conduct it. This would help close the divergence between the social ideology and the technological application of peer review.

***4.4.1 Future avenues of research.*** Rigorous, evidence-based research on peer review itself is surprisingly lacking across many research domains, and would help to build our collective understanding of the process and guide the design of ad-hoc solutions ( Bornmann & Daniel, 2010b; Bruce *et al.*, 2016; Rennie, 2016; Jefferson *et al.*, 2007). Such evidence is needed to form the basis for implementing guidelines and standards at different journals and research communities, and making sure that editors, authors, and reviewers hold each other reciprocally accountable to them. Further research should also focus on the challenges faced by researchers from peripheral nations, particularly for those who are non-native English speakers, and increase their influence as part of the globalization of research ( Fukuzawa, 2017; Salager-Meyer, 2008; Salager-Meyer, 2014). The scholarly publishing industry could help to foster such research into evidence-based peer review, by collectively sharing its data on the effectiveness of different peer review processes and systems, the measurement itself of which is still a problematic issue. Their incentives here are to help improve the process through rigorous, quantitative analysis, and also to help manage their own reputations ( Lee & Moher, 2017; Squazzoni *et al.*, 2017a; Squazzoni *et al.*, 2017b). Some progress is already being made on this front, coming from across a range of stakeholder groups. This includes:

1. A new journal, *Research Integrity and Peer Review*, published by BioMed Central to encourage further study into the integrity of research publication ( researchintegrityjournal.biomedcentral.com/);
2. The International Congress on Peer Review and Scientific Publication, which aims to encourage research into the quality and credibility of peer review ( peerreviewcongress.org/index.html;
3. The PEERE initiative, which has the objectives of improving the efficiency, transparency and accountability of peer review ( peere.org/).

While we briefly considered peer review in the context of some non-text products here (Section 3.9), there is clear scope for further discussion of the diverse applications of peer review. The utility of peer review for research grant proposals would be a fruitful avenue for future work, given that here it is less about providing feedback for authors, and more about making assessments of research quality. There are different challenges and different potential solutions to consider, but with some parallels to that discussed in the present manuscript. For example, how does the role of peer review for grants change for different applicant demographics in a time when funding budgets are, in some cases, being decreased, but in concert with increasing demand and research outputs.

One further aspect that we did not examine in detail is the use of instant messaging services, like *Slack* or *Gitter*. These are widely used for project communication and operate analogous to a real-time collaboration system with instantaneous and continuous “peer review”. While such activities can be used to supplement other hybrid platforms, as an independent or stand-alone mode of peer review, the concept is quite distant from the other models that have been discussed here (e.g., in terms of whether such messages are available in public, and for how long).

## 5 Conclusions

If the current system of peer review were to undergo peer review, it would undoubtedly achieve a “revise and resubmit” decision. As Smith (2010) succinctly stated, “we have little or no evidence that peer review ‘works,’ but we have lots of evidence of its downside”. There is further evidence to show that even the fundamental roles and responsibilities of editors, as those who manage peer review, has little consensus ( Moher *et al.*, 2017), and that tensions exist between editors and reviewers in terms of congruence of their responsibilities ( Chauvin *et al.*, 2015). These dysfunctional issues should be deeply troubling to those who hold peer review in high regard as a “gold standard”.

In this paper, we have presented an overview of what the key features of a hybrid, integrated peer review and publishing platform might be and how these could be combined. These features are embedded in research communities, which can not only set the rules of engagement but also form the judge, jury, and executioner for quality control, moderation, and certification. The major benefit of such a system is that peer review becomes an inherently social and community-led activity, decoupled from any journal-based system. We see adoption of existing technologies as motivation to address the systemic challenges with reviewer engagement and recognition. In our proposal, the abuse of power dynamics has the potential to be diminished or entirely alleviated, and the legitimacy of the entire process is improved. The “Principle of Maximum Bootstrapping” outlined by Kelty *et al.* (2008) is highly congruent with this social ideal for peer review, where new systems are based on existing communities of expertise, quality norms, and mechanisms for review. Diversifying peer review in such a manner is an intrinsic part of a system of reproducible research ( Munafò *et al.*, 2017). Making use of persistent identifiers such as *DataCite*, *CrossRef*, and *ORCID* will be essential in binding the social and technical aspects of this to an interoperable, sustainable and open scholarly infrastructure ( Dappert *et al.*, 2017).

However, we recognize that any technological advance is rarely innocent or unbiased, and while Web 2.0 technologies open up the possibility for increased participation in peer review, it would still not be inherently democratic ( Elkhatib *et al.*, 2015). As Belojevic *et al.* (2014) remark, when considering tying reputation engines to peer review, we must be aware that this comes with implications for values, norms, privilege and bias, and the industrialization of the process ( Lee *et al.*, 2013). Peer review is socially and culturally embedded in scholarly communities and has an inherent diversity in values and processes, which we must have a deep awareness of and appreciation for. The major challenge that remains for any future technological advance in peer review will be how it captures this diversity, and embeds this in its social formation and operation. Therefore, there will be difficulties in defining the boundaries of not just peer review types, but the boundaries of communities themselves, and how this shapes any community-led process of peer review.

Academics have been entrusted with an ethical imperative towards accurately generating, transforming, and disseminating new knowledge through peer review and scholarly communication. Peer review started out as a collegial discussion between authors and editors. Since this humble origin, it has vastly increased in complexity and become systematized and commercialized in line with the neo-liberal evolution of the modern research institute. This system is proving to be a vast drain upon human and technical resources, due to the increasingly unmanageable workload involved in scholarly publishing. There are lessons to be learned from the Open Access movement, which started as a set of principles by people with good intentions, but was subsequently converted into a messy system of mandates, policies, and increased costs that is becoming increasingly difficult to navigate. Commercialization has inhibited the progress of scholarly communication, and can no longer keep pace with the generation of new ideas in a digital world.

Now, the research community has the opportunity to help create efficient and socially-responsible systems of peer review. The history, technology, and social justification to do so all exist. Research communities need to embrace the opportunities gifted to them and work together across stakeholder boundaries (e.g., with research funders, libraries and professional communicators) to create an optimal system of peer review aligned with the diverse needs of non-independent research communities. By decoupling peer review, and with it scholarly communication, from commercial entities and journals, it is possible to return it to the core principles upon which it was founded as a community-based process. Through this, knowledge generation and access can become a more democratic process, and academics can fulfil the criteria that have been entrusted to them as creators and guardians of knowledge.
