# Supplementary material for: A multi-disciplinary perspective on emergent and future innovations in peer review
Source: F1000Res. 2017 Nov 29;6:1151. Originally published 2017 Jul 20. [Version 3] doi: 10.12688/f1000research.12037.3 (PMC5686505; doi:10.12688/f1000research.12037.3)
Supplement: Peer review history

# Peer review: Referee response for version 2

**DOI:** 10.5256/f1000research.14133.r27486

**Published:** 2017-11-13

**Authors:** Virginia Barbour

**Affiliations:** 1 Queensland University of Technology (QUT), Brisbane, Qld, Australia

General comments

 On reading this again, I still think it is very long and overly repetitive (for example the various types of open peer review are discussed in a number of sections) and the paper would benefit from substantial consolidation of key topics. I recognize, however, that the authors are unlikely to be able to substantially change it now, given the large and diverse authorship. I note that a number of changes were made, though as far I can see the authors didn’t respond specifically to all the points I made, hence a number of my previous comments still stand. I'll leave it up to the authors if they respond to these comments.

 1.0.1 Methods

 Thanks for clarifying that there was not a systematic review process here. I think it would be helped if the limitation of the approach taken was noted within the methodology (for example that there was no formal search strategy undertaken with specific keywords). It is clearer that the paper does express a multitude of perspectives, rather than being definitive.

 The authors argue against considering peer review as a “gold standard” but again on re-reading it I think the overall tone of the article may actually risk perpetuating the myth that peer review is some sort of panacea, rather than essentially a QC process. Anything that helps in demystification of the process  would be helpful in encouraging a healthy debate in this area.

 I agree with this statement in the conclusions *“Now, the research community has the opportunity to help create an efficient and socially-responsible system of peer review.”*, though I think it is more likely there will be a multitude of systems that we should be looking to support.

**Specific comments**

 There are a number of typos and sentences that need clarification - I have noted those I found.

 Italics are used when quoting from the paper

 Introduction

*However, peer review is still often perceived as a “gold standard” (e.g., D’Andrea & O’Dwyer (2017); Mayden (2012)), despite the inherent diversity of the process and never intended to be used as such. *There seems to be text missing from this sentence

*By allowing the process of peer review to become managed by a hyper-competitive industry, developments in scholarly publishing have become strongly coupled to the transforming nature of academic research institutes.*  I am not sure what this sentence means.

 1.3

*In response to this, initiatives like the EQUATOR network ( equator-network.org) have been important to improve the reporting of research and its peer review according to standardised criteria. Another response has been COPE, the Committee on Publication Ethics (* *publicationethics.org* *), was established in 1997 to address potential cases of abuse and misconduct during the publication process. (specifically regarding author misconduct), and later created specific guidelines for peer review.*

 I don’t think that EQUATOR was set up as a result of specific criticisms about peer review – it was set up to address poor reporting of papers, and COPE certainly wasn’t set up to address peer review. COPE did later produce guidelines and other resources for peer reviewers, which have been recently updated – see https://publicationethics.org/peerreview

*A popular editorial in The BMJ stated that made some quiter serious* (typo “quiter”)

 2.2.1 Most statistical reviewers are paid. This is not mentioned in the text

 2.4.2 *The Committee on Publication Ethics (COPE) could be used as the basis for developing formal mechanisms adapted to innovative models of peer review, including those outlined in this paper. * 

 COPE does advise on new peer review models as appropriate to ethics cases so I am not sure what is meant here.

 2.5.1

*Papers on digital repositories are cited on a daily basis and much research builds upon them, although they may suffer from a stigma of not having the scientific stamp of approval of peer review*

 Should specify that this section is for preprint repositories

 2.5.2

 It’s a shame that clinical trial registration is not discussed more, though I appreciate it being added. It’s an interesting example of how innovations in one specialty often are not fully appreciated in another (until that specialty reinvents it for itself!).

I have read this submission. I believe that I have an appropriate level of expertise to confirm that it is of an acceptable scientific standard.

# Peer review: Referee response for version 2

**DOI:** 10.5256/f1000research.14133.r27485

**Published:** 2017-11-10

**Authors:** David Moher

**Affiliations:** 1 Centre for Journalology, Clinical Epidemiology Program, Ottawa Hospital Research Institute, Ottawa, ON, Canada

The revisions are good. The paper is now more mature. I am comfortable accepting it for indexing.

 I noted a typo - 2.4.2 The Rodriguez-Bravo et al should have a "In" prior to "Press".

I have read this submission. I believe that I have an appropriate level of expertise to confirm that it is of an acceptable scientific standard.

# Peer review: Referee response for version 1

**DOI:** 10.5256/f1000research.13023.r24355

**Published:** 2017-08-14

**Authors:** Virginia Barbour

**Affiliations:** 1 Queensland University of Technology (QUT), Brisbane, Qld, Australia

Thank you for asking me to review this paper.

 Ironically, but perhaps not surprisingly, this was quite a hard paper to peer review and I don’t claim that this peer review does anything more than provide one (non-exhaustive) opinion on this paper. My views on peer review, which have formed over more than 15 years of being involved in editing and managing peer review will have coloured my peer review here.

 I think it's useful to regard all journal processes, which includes peer review, as components of the QC which begins with checking for basics such as the presence of ethics statements or trial registration, or the use of  reporting guidelines for example, through to in depth methodological review. I don't think that any of the parts of the system of QC, including peer review, are perfect but the system is one component of attempting to ensure reproducibility, itself a core role of journals. The very basic functions of QC  are often not given enough emphasis, though they are going to become more important as, for example, other types of publication such as  preprints increase in popularity.

**General Comments**

 This is a wide ranging, timely paper and will be a useful resource.

 My main comment is that this is a mix of opinion, review, and thought experiment of future models. While all of these are needed in this area, for the review part of the paper, it would be much strengthened with a description of the methodology used for the review, including databases searched for information and keywords used to search, etc.

 The paper is very long and there is a substantial amount of repetition. I think the introduction in particular could be much shortened - especially as it contains a lot of opinion, and repetition of issues dealt with elsewhere in the paper.

 The language of the paper is also quite emotive in places and though I would personally agree with some of the sentiments I don't think they are helpful in making the authors’ case eg in Table 2  assessment of pre publication peer review is listed as *Non-transparent,impossible to evaluate,biased, secretive, exclusive*

 Or *The entrenchment of the ubiquitously practiced and much more favored traditional model (which, as noted above, is also diverse) is ironically non-traditional, but nonetheless currently revered*.  

 I think it worth reviewing the language of the paper with that in mind.

 Although it arises in a number of places I don’t feel the authors address fully the complexity of interdisciplinary differences. The introduction would have been a good place to set this down.

 There is no mention of initiatives such as EQUATOR which have been important in improving reporting of research and its peer review. http://www.equator-network.org/

 I was surprised to see very little discussion of the problems associated with commenting - especially of tone - that can arise on anonymous or pseudonymous sites such as Pubpeer and reddit.

 There was no discussion of post publication reviews which originate in debates on twitter. There have been some notable examples of substantial peer review happening - or at least beginning there eg that on arsenic life ^1^.

 There are quite a few places where initiatives are mentioned but not referenced or hyperlinked. eg *Self Journal of Science.*

**Specific comments**

 Introduction

 I would take issue with the term “ *gold standard*”. In my view many of the issues arising from peer review are that it is held to a standard that was never intended for it.

 Introduction paragraph 2 - where PLOS is mentioned here it should be replaced by PLOS ONE - the other journals from PLOS have other criteria for review. I am surprised that PLOS ONE does not get more of a mention in how much of a shift it represent in its model of uncoupling objective from subjective peer review, and how it led to the entire model for mega journals.

 1.1.1 *“* *The purpose of developing peer reviewed journals became part of a process to deliver research to both generalist and specialist audiences, and improve the status of societies and fulfil their scholarly missions”*

 I think it is worth noting that another function of peer review at journals was that it was part of earliest attempts of ensuring reproducibility - which is of course a very hot topic nowadays but in fact has its roots right back to when experiments were first described in journals.

*“From these early developments, the process of independent review of scientific reports by acknowledged experts gradually emerged. However, the review process was more similar to non-scholarly publishing, as the editors were the only ones to appraise manuscripts before printing”*

 There is a misconception here, which I think is quite common. In the vast majority of cases editors are also peers, and may well be “acknowledged experts” - in fact certainly will be at society journals. The distinction between editors and peer reviews can be a false one with regard to expertise.

*1.1.2 where publishers call upon external specialists to validate journal submissions.*

 It is important to note that it is editors who manage review processes. Publisher are largely responsible for the business processes; editors for the editorial processes.

*By allowing the process of peer review to become managed by a hyper-competitive industry, developments in scholarly publishing have become strongly coupled to the transforming nature of academic research institutes.  “ These have evolved into internationally competitive businesses that strive for quality through publisher-mediated journals by attempting to align these products with the academic ideal of research excellence (* *Moore et al., 2017* *)”*

 I am not sure what is meant by “these” in this second sentence, nor what is meant by a “publisher-mediated  journal”. Virtually all journals have a publisher - even small academic-led ones.

*1.1.3  This practice represents a significant shift, as public dissemination was decoupled from a traditional peer review process, resulting in increased visibility and citation rates (* *Davis & Fromerth, 2007* *;* *Moed, 2007* *).*

 Many papers posted on arxiv.org do go on to be published in peer reviewed journals. Are these references referring to  increased citation of the preprints or the version published in a peer reviewed journal?

*The launch of Open Journal Systems (* *openjournalsystems.com* *; OJS) in 2001 offered a step towards bringing journals and peer review back to their community-led roots.*  

 The jump here is odd. OJS actually can support a number of models of peer review, including a traditional model of peer review, just on a low cost open source platform, not a commercial one. The innovation here is the technology.

*Digital-born journals, such as PLOS ONE, introduced commenting on published papers.*  

 Here the reference should be to all of PLOS as commenting was not unique to PLOS ONE. However, the better example of commenting is the BMJ which had a vibrant paper letters page which it transformed very successfully to its rapid responses - and it remains the  journal that has had most success http://www.bmj.com/rapid-responses.

*Other services, such as Publons, enable reviewers to claim recognition for their activities as referees.*

 Originally Academic Karma http://academickarma.org/ had a similar purpose though now it has a different model - facilitating peer review of preprints.

 Figure 2

 PLOS ONE and ELife should be added to this timeline. Elife’s collaborative peer review model is very innovative. I am not sure why Wikipedia is in here.

*1.3* *One consequence of this is that COPE, the Committee on Publication Ethics (* *publicationethics.org* *), was established in 1997 to address potential cases of abuse and misconduct during the publication process.*

 COPE was first established because of issues related to author misconduct which had been identified by editors. Though it does now have a number of cases relating to peer review , the guidelines for peer review came much later and peer review was not an early focus.

*Taken together, this should be extremely worrisome, especially given that traditional peer review is still viewed almost dogmatically as a gold standard for the publication of research results, and as the process which mediates knowledge dissemination to the public.*

 I am not sure I would agree. Every person I know who works in publishing accepts that peer review is an imperfect system and that there is room for rethinking the process. Sense about Science puts it well in its guide: ” *Just as a washing machine has a quality kite-mark, peer review is a kind of quality mark for science. It tells you that the research has been conducted and presented to a standard that other scientists accept. At the same time, it is not saying that the research is perfect (nor that a washing machine will never break down).* http://senseaboutscience.org/wp-content/uploads/2016/09/peer-review-the-nuts-and-bolts.pdf

 Table 2.

 Note that quite a few of these approaches can co-exist. Under post publication commenting PLOS ONE should be PLOS. BMJ should be added here.

 1.4 Quite a lot of subscription journals do reward reviewers by providing free subscriptions to the journal - or OA journals provide discounts on APCs (including F1000). Furthermore, some reviewers are paid, especially statistical reviewers.

 2.2.2 *Hence, this [Wiley] survey could represent a biased view of the actual situation. * 

 I’d like to see evidence to support this statement.

*2.2.3  The idea here is that by being able to standardize peer review activities, it becomes easier to describe, attribute, and therefore recognize and reward them*

 I think the idea is to standardise the description of peer review, not the activity itself. Please clarify.

 2.4.2. *Either way, there is little documented evidence that such retaliations actually occur either commonly or systematically. If they did, then publishers that employ this model such as Frontiers or BioMed Central would be under serious question, instead of thriving as they are. * 

 This sentence seems to be in contradiction to the phrase below:

*In an ideal world, we would expect that strong, honest, and constructive feedback is well received by authors, no matter their career stage. Yet, it seems that this is not the case, or at least there seems to be the very real perception that it is not, and this is just as important from a social perspective. Retaliations to referees in such a negative manner represent serious cases of academic misconduct*

*2.5.1. This process is mediated by ORCID for quality control, and CrossRef and Creative Commons licensing for appropriate recognition. They are essentially equivalent to community-mediated overlay journals, but with the difference that they also draw on additional sources beyond pre-prints.*

 This is an odd description. In what way does ORCID mediate for quality control?

*2.5.2 Two-stage peer review and Registered Reports.*

 Registration of clinical trials predated registered reports by a number of years and it would be useful to include clinical trial registration in this section.

*3 Potential future models*  

 NB I didn’t review this section in detail.

*3.5 as was originally the case with Open Access publishing,*

 The perception of low quality in OA was artificially perpetuated by traditional publishers more than anything else - it was not inherent to the process.

 3.5 Wikipedia and PLOS Computational Biology collaborated in a novel peer review experiment which would be worth mentioning  - see http://journals.plos.org/ploscompbiol/article?id=10.1371/journal.pcbi.1002446 ^2^.

 3.9.2 Data peer review. This is  a vast topic and there are many initiatives in this area, which are not really discussed at all. I would suggest this section should come out - especially as earlier on it is noted that the paper focuses mainly on peer review of traditional papers. I would also suggest taking out the parts on OER and books.

I have read this submission. I believe that I have an appropriate level of expertise to confirm that it is of an acceptable scientific standard, however I have significant reservations, as outlined above.

## References

[1] YeoSKLiangXBrossardDRoseKMKorzekwaKScheufeleDAXenosMA: The case of #arseniclife: Blogs and Twitter in informal peer review. *Public Underst Sci*.2016; 10.1177/0963662516649806 10.1177/0963662516649806 27229853

[2] WodakSMietchenDCollingsARussellRBourneP: Topic Pages: PLoS Computational Biology Meets Wikipedia. *PLoS Computational Biology*.2012;8(3) : 10.1371/journal.pcbi.1002446 10.1371/journal.pcbi.1002446 PMC331544722479174

# Peer review: Referee response for version 1

**DOI:** 10.5256/f1000research.13023.r24353

**Published:** 2017-08-07

**Authors:** David Moher

**Affiliations:** 1 Centre for Journalology, Clinical Epidemiology Program, Ottawa Hospital Research Institute, Ottawa, ON, Canada

This manuscript is a herculean effort and enjoyable read. I learned lots, (and I think the paper will be a good resource for anybody interested in the field of peer review) which for me is usually a good sign of a paper’s worth.

 The authors report on many aspects of peer review and devote considerable attention to some challenges in the field and the enormous innovation the field is witnessing.

 I think the paper can be improved:

 1. It is missing a Methods section. It was unclear to me whether the authors conducted a systematic review or whether they used a snowballing technique (starting with seed articles) to identify the content discussed in the paper? Did the authors search electronic databases (and if so which ones?) What were their search strategies and/or did they rely on their own file drawers? Are all the peer review innovations/systems/approaches identified by the authors discussed or did they only discuss some (i.e., was a filter applied?)? With a focus on reproducibility I think the authors need to document their methods.

 2. I think the authors missed an important opportunity to discuss more deeply the need for evidence with all the current and emerging peer review systems (the authors reference Rennie 2016 ^1^ in their conclusions. I think the evidence argument needs to be made more strongly in the body of the paper). I do not think the paper is strong enough regarding the large swaths of the peer review processes (current and innovations) for which there is no evidence ^2^ and it is difficult to gain access to peer reviews to better understand their processes and effectiveness – open the black box of peer review ^3^.

 3. There is limited data to inform us about several of the current peer review systems and innovations. In clinical medicine new drugs do not simply enter the market. They need undergo a rigorous series of evaluations, typically randomized trials prior to approval. Shouldn’t we expect something similar for peer review in the marketplace? It seems to me that any peer review process/innovation in development or released should have an evaluation (experimental, whenever possible) component integrated into it. Without evaluation we will miss the opportunity to generate data as to the effectiveness of the different peer review systems and processes. Research is central to getting a better understanding of peer review. It might useful for the authors to mention the existence of some groups/outlets committed to such research – PEERE (http://www.peere.org/) and the International Congress on Peer Review and Scientific Publication (http://www.peerreviewcongress.org/index.html). There is also a new journal committed to publishing peer review research ( https://researchintegrityjournal.biomedcentral.com/).

 4. In section 1.3 of the paper the authors could add (or replace) Jefferson 2002 ^4^ with Bruce ^5^. The Bruce paper is also important for two additional reasons not adequately discussed in the paper: how to measure peer review and optimal designs for assessing the effects of peer review. 

 Concerning measurement of peer review, there is accumulating evidence that there is little agreement as to how best to measure it. Unlike clinical medicine where there is a growing recognition of the need for core outcome set assessments (http://www.comet-initiative.org/) across all studies within specific content areas (e.g., atopic eczema/dermatitis clinical trials) we have not yet developed such an approach for peer review. Without a core outcome set for measuring peer review it will continue to be difficult to know what components of peer review researchers are trying to measure.

 Similarly, without a core outcome set it will be difficult to aggregate estimates of peer review across studies (i.e., to do meaningful systematic reviews on peer review).

 Concerning the second point – there is little agreement as to an optimal design to evaluate the effectiveness of peer review. This is a critical issue to remedy in any effort to assess the effectiveness of peer review.

 5. The paper assumes (at least that’s how I’ve interpreted it – the paper is silent on this issue) that peer reviewers are all similarly proficient in peer reviewing. There is little training for peer reviewers (new efforts by some organizations such as Publons Academy are trying to remedy this). I started my peer-reviewing career without any training, as did many of my colleagues. If we do not train peer reviewers to a minimum globally accepted standard we will fail to make peer review better.  

 6. Peer review does not function in a vacuum. The larger ecosystem includes other players’ most notably scientific editors. There is little discussion in the paper about this relationship and its potential (dys)function ^6^.

 7. In section 2.2.1 you could also add at least one journal has an annual prize for peer reviewing (Journal of Clinical Epidemiology – JCE Reviewer Award: http://www.jclinepi.com/)

 8. In the competing interests section of the paper it indicates that the first author works at ScienceOpen although the affiliation given in the paper is Imperial College London. Is this a joint appointment? Clarification is needed. A similar clarification is required for TRH.

I have read this submission. I believe that I have an appropriate level of expertise to confirm that it is of an acceptable scientific standard, however I have significant reservations, as outlined above.

## References

[1] RennieD: Let’s make peer review scientific. *Nature*.2016;535(7610) : 10.1038/535031a 31-33 10.1038/535031a 27383970

[2] GalipeauJMoherDCampbellCHendryPCameronDWPalepuAHébertPC: A systematic review highlights a knowledge gap regarding the effectiveness of health-related training programs in journalology. *J Clin Epidemiol*.2015;68(3) : 10.1016/j.jclinepi.2014.09.024 257-65 10.1016/j.jclinepi.2014.09.024 25510373

[3] LeeCJMoherD: Promote scientific integrity via journal peer review data. *Science*.2017;357(6348) : 10.1126/science.aan4141 256-257 10.1126/science.aan4141 28729501

[4] JeffersonTWagerEDavidoffF: Measuring the quality of editorial peer review. *JAMA*.2002;287(21) :2786-90 1203891210.1001/jama.287.21.2786

[5] BruceRChauvinATrinquartLRavaudPBoutronI: Impact of interventions to improve the quality of peer review of biomedical journals: a systematic review and meta-analysis. *BMC Med*.2016;14(1) : 10.1186/s12916-016-0631-5 85 10.1186/s12916-016-0631-5 27287500PMC4902984

[6] ChauvinARavaudPBaronGBarnesCBoutronI: The most important tasks for peer reviewers evaluating a randomized controlled trial are not congruent with the tasks most often requested by journal editors. *BMC Med*.2015;13: 10.1186/s12916-015-0395-3 158 10.1186/s12916-015-0395-3 26141137PMC4491236
